# Positive regulation of meiotic DNA double-strand break formation by activation of the DNA damage checkpoint kinase Mec1(ATR)

**DOI:** 10.1098/rsob.130019

**Published:** 2013-07

**Authors:** Stephen Gray, Rachal M. Allison, Valerie Garcia, Alastair S. H. Goldman, Matthew J. Neale

**Affiliations:** 1 MRC Genome Damage and Stability Centre, School of Life Sciences, University of Sussex, Brighton BN1 9RQ, UK; 2 Department of Molecular Biology and Biotechnology, University of Sheffield, Sheffield S10 2TN, UK

**Keywords:** meiosis, checkpoint, recombination, Mec1 ATR, Tel1 ATM, Spo11

## Abstract

During meiosis, formation and repair of programmed DNA double-strand breaks (DSBs) create genetic exchange between homologous chromosomes—a process that is critical for reductional meiotic chromosome segregation and the production of genetically diverse sexually reproducing populations. Meiotic DSB formation is a complex process, requiring numerous proteins, of which Spo11 is the evolutionarily conserved catalytic subunit. Precisely how Spo11 and its accessory proteins function or are regulated is unclear. Here, we use *Saccharomyces cerevisiae* to reveal that meiotic DSB formation is modulated by the Mec1(ATR) branch of the DNA damage signalling cascade, promoting DSB formation when Spo11-mediated catalysis is compromised. Activation of the positive feedback pathway correlates with the formation of single-stranded DNA (ssDNA) recombination intermediates and activation of the downstream kinase, Mek1. We show that the requirement for checkpoint activation can be rescued by prolonging meiotic prophase by deleting the *NDT80* transcription factor, and that even transient prophase arrest caused by Ndt80 depletion is sufficient to restore meiotic spore viability in checkpoint mutants. Our observations are unexpected given recent reports that the complementary kinase pathway Tel1(ATM) acts to inhibit DSB formation. We propose that such antagonistic regulation of DSB formation by Mec1 and Tel1 creates a regulatory mechanism, where the absolute frequency of DSBs is maintained at a level optimal for genetic exchange and efficient chromosome segregation.

## 2. Introduction

To prevent the chromosome copy number doubling during each reproductive cycle, sexually reproducing organisms create gametes containing half the chromosome copy number of their parents. Halving the chromosome copy number occurs during meiosis—a specialized pair of sequential nuclear divisions that first segregates homologous chromosomes, then sister chromatids. In many organisms, accurate segregation of homologous chromosomes during meiosis I requires the formation and repair of numerous DNA double-strand breaks (DSBs) distributed across the genome. DSB repair uses homologous recombination to drive interaction and genetic exchange between homologous chromosomes, which facilitates accurate chromosome disjunction at anaphase I. Much of our detailed understanding of the mechanisms of meiotic recombination comes from studies performed in the budding yeast *Saccharomyces cerevisiae* [1].

In *S. cerevisiae*, meiotic DSB formation requires the function of 10 proteins, of which Spo11 is the catalytic subunit. The precise function of the nine accessory proteins is unclear, as are the detailed mechanisms that govern the frequency and distribution of Spo11–DSB formation across the genome. Spo11 shares similarity to a type-II topoisomerase (Top6A) from archaea and initiates DSB formation via a transesterification reaction that coordinately severs the DNA backbone on both strands, leaving Spo11 monomers covalently attached to the 5′ ends of the DSB (reviewed in [2]).

Spo11–DSBs are processed nucleolytically by the Mre11, Sae2 and Exo1 proteins, creating DSBs with extended 3′-ending single-stranded termini [3–18], which are rapidly bound by Replication Protein A [19]. In the absence of Mre11 nuclease activity or of the accessory factor, Sae2, Spo11 remains covalently bound to DSB ends [8,11–14,18,20,21]. In these circumstances, repair of the DSBs is impossible and cells undergo a catastrophic meiotic nuclear division that generates fragmented chromosomes [4–6,18].

In wild-type cells, the ssDNA ends are used in a homology search by the eukaryotic RecA orthologues, Rad51 and Dmc1 [22,23], creating intermolecular interactions between homologous chromosome pairs. In the absence of Dmc1, DSBs accumulate with extended ssDNA ends due to failed repair [22]. The accumulation of ssDNA elicits a strong checkpoint response, causing cells to arrest in late prophase I [22,24], at least in part due to inactivation of the meiotic transcription factor, Ndt80 [25]. In mammals, similar recombination failure leads to programmed cell death [26,27]. The checkpoint response depends on activation of orthologues of the mammalian phosphoinositide-3-kinase-related kinase (PIKK) ATR, the RAD9-RAD1-HUS1 (9-1-1) checkpoint clamp complex and the RAD17 clamp loader [28–31]. In budding yeast, these proteins are, respectively, named Mec1, Rad17, Mec3, Ddc1 and Rad24 [32,33]. Such activation leads to persistent phosphorylation of Mek1 [34,35], a meiosis-specific paralogue of the Rad53 kinase (CHK2 in mammalian cells [36–39]).

Removing the function of checkpoint proteins causes entry into the meiotic divisions of mutants that accumulate unrepaired DSBs, leading to cell death [24]. Interestingly, budding yeast cells, which possess mutations in the checkpoint proteins, actually undergo a delayed DSB-dependent meiosis I division, suggesting that checkpoint proteins are directly involved in the DNA repair and recombination process itself [40]. Indeed, checkpoint mutants have increased rates of ectopic (also called, non-allelic) recombination [41], whereas mutation of Mek1 abolishes repair partner choice entirely, with most or all DSBs now competent to repair using the sister chromatid (rather than the homologous chromosome) as repair template [42–44].

Recently, it has been shown that mutation of another PIKK kinase, ATM, results in increased DSB formation in mammals and in flies [45,46], and both Mec1(ATR) and Tel1(ATM) are necessary in budding yeast to reduce the likelihood that more than one DSB arises per chromatid quartet at a given chromosomal locus [47]. Here, we reveal a novel role for the Mec1 pathway in promoting DSB formation when the catalytic activity of Spo11 is compromised. Our results suggest that both positive and negative feedback loops influence the rate of DSB formation, creating a regulated system with optimal levels of recombination initiated.

## 3. Results

### 3.1. Loss of Rad24/Mec1(ATR) checkpoint activity causes a synergistic reduction in double-strand break signals in hypomorphic *spo11-HA* strains

**Figure 1.**
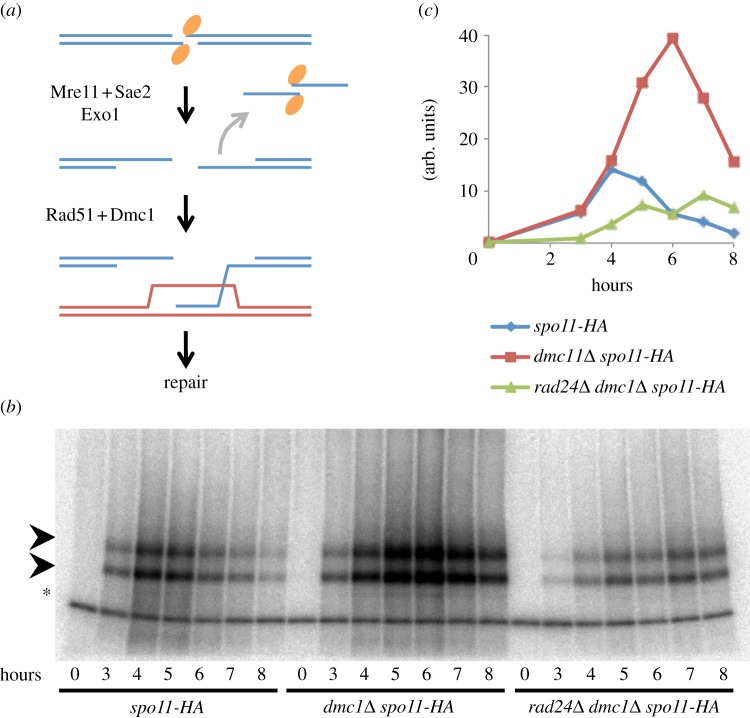
Levels of Spo11-oligonucleotides are increased in the recombination-defective *dmc1Δ* mutant but decreased when combined with the *rad24Δ* checkpoint mutant. (*a*) Meiotic recombination is initiated by DSBs created by Spo11 (orange ellipses), which are nucleolytically processed by Mre11, Sae2 and Exo1 generating transient Spo11-oligonucleotide complexes and ssDNA flanking the DSB site. DSB repair uses Dmc1 and Rad51-dependent recombination between homologous chromosomes, which facilitates their pairing and segregation at anaphase I. (*b*) Spo11-oligonucleotide complexes were immunoprecipitated from whole cell extracts of synchronous cultures of the indicated strains and timepoints after meiotic induction, 3′-labelled with α-^32^P dCTP using TdT and resolved by SDS-PAGE. Spo11-oligonucleotides are marked by an arrowhead. Asterisk indicates non-specific TdT labelling. (*c*) Abundance of Spo11-oligonucleotide complexes was quantified using a phosphorimager and ImageGauge software.

In each budding yeast meiotic cell, accurate reductional segregation of homologous chromosomes at the first nuclear division involves the formation and repair of approximately 160 Spo11–DSBs [48,49]. The first step in the repair of Spo11–DSBs is their nucleolytic processing by Mre11 and Sae2, creating covalent Spo11-oligonucleotide complexes (Spo11-oligos; figure 1*a* [11,18,48]). Spo11-oligos are detected by immunopurifying Spo11 and radiolabelling the ends of the covalently attached DNA [50]. The absolute abundance of Spo11-oligo complexes can be used to estimate total DSB formation. Spo11–DSBs trigger transient activation of the DNA damage checkpoint response machinery (DDR [24])—a state that persists in recombination-defective mutants such as *dmc1Δ* [22]. During our studies, we were intrigued to discover that while the abundance of Spo11-oligo complexes was increased in *dmc1Δ* strains (where DSB repair is abolished, and DSBs accumulate [22]), such an increase was absent when the DDR was inactivated (*rad24Δ*; figure 1*b,c*).

Our observations suggest a link between checkpoint activity and Spo11-oligo abundance. However, one limitation of our method is that it makes use of a *SPO11-HA3-His6* allele (henceforth referred to as *spo11-HA*) to enrich for Spo11-oligo complexes. This C-terminal epitope tag is reported to moderately reduce the catalytic activity of Spo11 (DSB formation drops by 11–50% compared with the untagged wild-type [51]). Because the reduction in Spo11-oligo abundance in *rad24Δ* *dmc1Δ* could be due to fewer total DSBs or to their faster turnover—perhaps due to changes in meiotic cell cycle progression caused by *rad24Δ*—we were interested to determine the abundance of DSBs in our panel of strains.

**Figure 2.**
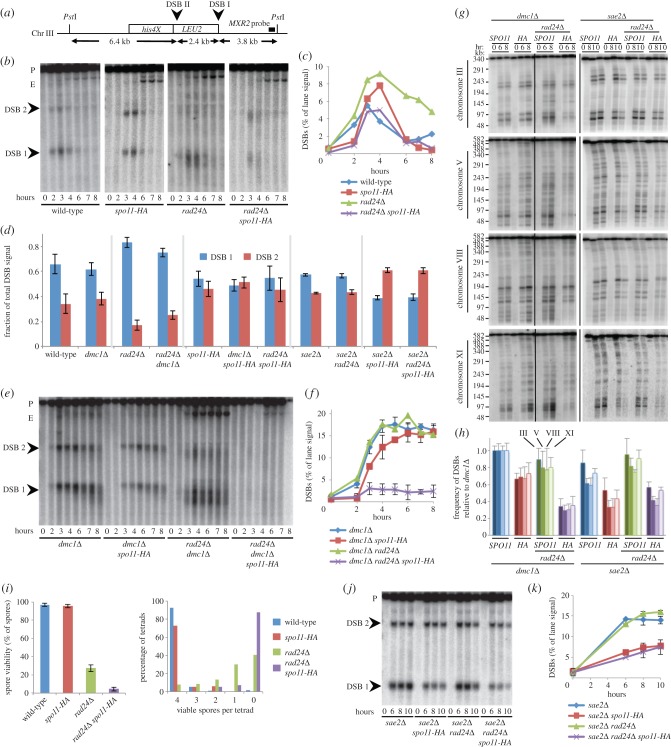
Synergistic reduction in meiotic DSBs in *rad24Δ spo11-HA* strains. (*a*) Physical map of the *HIS4::LEU2* region, including *Pst*I restriction sites, DSB sites and location of *MXR2* probe. (*b*,*e*,*j*) Genomic DNA was isolated at the indicated time points from synchronous cultures of the indicated strains, digested with *Pst*I, fractionated on a 0.7% agarose gel, transferred to nylon membrane and hybridized with the *MXR2* probe. Arrowheads indicate DSB signals; P, parental band; E, ectopic recombinant (see electronic supplementary material, figure S2). (*c*,*f*,*k*) Quantification of the total DSB signal (DSB 1+DSB 2) shown in (*b*,*e*,*j*) plotted as a percentage of total lane signal. (*d*) Proportion of DSB signal present in DSB 1 and DSB 2 for the indicated strains. (*g*) Intact chromosomal DNA was isolated at the indicated time points from synchronously sporulating meiotic cultures, separated by PFGE, transferred to a nylon membrane and sequentially hybridized using a radiolabelled fragment of a gene close to the left telomere of each chromosome (chr III = *CHA1*; chr V = *RMD6*; chr VIII = *CBP2*; chr XI = *JEN1*). Intact, full-length chromosomes migrate near the top of the gel. DSB signals appear as the shorter, faster-migrating bands/molecules present in the 6–10 h timepoints. (*h*) Quantification of total DSB signals in each lane expressed as a fraction of total lane signal. Measured signals were adjusted to account for the likelihood of multiple DSBs occurring on the same molecule (see material and methods). Because the absolute frequency of DSB formation varies with chromosome, calculated average frequencies (±s.d.) are plotted relative to *dmc1Δ* to aid easier strain-to-strain comparison. (*i*) Spore viability displayed as total viability (left) or separated according to number of viable spores per tetrad (right). Error bars (left) are 95% confidence limits.

We began by assessing DSB formation at the well-characterized *HIS4*::*LEU2* reporter locus on chromosome III [52] in synchronized meiotic cultures, where Spo11 was either HA-tagged or untagged. At *HIS4::LEU2*, there are two prominent DSB sites flanking the *LEU2* insertion (figure 2*a*). In the untagged strain (*SPO11*^+^), DSB signals peaked at approximately 6% of the total lane signal (figure 2*c*), with more of the DSBs arising at site 1 (figure 2*d*). Contrary to our expectation, total DSB signals were not reduced by the Spo11-HA tag (figure 2*c*). However, the distribution was altered such that now a similar proportion of DSBs was detected at site 1 and site 2 (figure 2*d*; see also [52]).

Accurately measuring DSB abundance from a culture of wild-type cells is complicated by overlapping kinetics of DSB formation and repair. To work around this issue, we remeasured the DSB frequency in strains similar to those we had used for the Spo11-oligo analysis, where the Dmc1 repair protein was mutated, causing DSBs to accumulate (figure 2*e*). In both the tagged and untagged Spo11 strains, DSBs accumulated to a similar extent, reaching approximately 15% and approximately 18% of total lane signal, respectively (figure 2*f*). As before, the distribution of events was altered by the HA-tag, such that the fractions of DSBs forming at site 1 and site 2 were now similar (figure 2*d*). A second strong hotspot region, *ARE1*, showed the same trend of relatively unchanged DSB frequency when the *spo11-HA* allele was present (see electronic supplementary material, figure S1*a*,*b*).

Next, we assessed DSB formation in the checkpoint-defective *rad24Δ* strain (figure 2*b–f*). Reduced *MEC1* checkpoint activity (as caused by mutations such as *rad24Δ*) results in rapid exonucleolytic hyper-resection of the DSB ends creating extensive 3′-ending ssDNA tails that cause DSB molecules to migrate much more rapidly and heterogeneously when separated by agarose electrophoresis [40]. At *HIS4::LEU2*, despite the signal arising in *rad24Δ* being more diffuse, DSBs remained readily detectable above lane background signals, peaking at 9% (figure 2*c*), slightly greater than wild-type cells, possibly due to a slight delay in DSB repair causing DSB signals to transiently accumulate to higher steady-state levels. In *rad24Δ* *dmc1Δ* cells, despite extensive hyper-resection, DSBs accumulated and persisted similar to *RAD24* *dmc1Δ*, reaching a maximum level of approximately 16–20% of the total DNA (figure 2*e*,*f*). Notably, in the *rad24Δ* and *rad24Δ* *dmc1Δ* backgrounds, barely any signal was observed at DSB site 2, resulting in a significantly altered ratio compared with *RAD24*^+^ cells (figure 2*d*). In addition, we observed increases in the frequency of ectopic recombination products in *rad24Δ* strains (see figure 2*b*,*e*; electronic supplementary material, figure S2*a*–*c*), as has been reported by others [41].

In contrast to the situation in *RAD24*^+^ cells, the combination of *spo11-HA* and *rad24Δ* resulted in a substantial reduction in DSB signal (figure 2*b*–*f*). DSBs were reduced to 50% of the *rad24Δ* level in *rad24Δ* *spo11-HA* cells (figure 2*b*,*c*), and to less than 25% of the *rad24Δ* *dmc1Δ* level in the *rad24Δ* *dmc1Δ* *spo11-HA* strain, where DSB signals were barely detected despite being expected to accumulate (figure 2*e*,*f*). These reductions in DSB abundance correlated with reduced abundance of ectopic recombination products (see electronic supplementary material, figure S2). Similar effects were observed at the *ARE1* locus (see electronic supplementary material, figure S1*b*). In *rad24Δ* *dmc1Δ* cells, despite extensive hyper-resection, DSBs accumulated to levels similar to both the *dmc1Δ* and *dmc1Δ* *spo11-HA* controls (see electronic supplementary material, figure S1*b*). By contrast, DSBs were undetectable in the *dmc1Δ* *rad24Δ* *spo11-HA* strain (see electronic supplementary material, figure S1*b*). This surprising reduction in DSB abundance was reproduced in two independently generated isolates of the same *rad24Δ* *dmc1Δ* *spo11-HA* genotype, indicating that the phenotype was not due to an underlying defect in the genotype of the strain nor a culture problem (data not shown).

To determine whether the synergistic reduction in DSB abundance observed in *rad24Δ* *spo11-HA* strains was a general phenomenon affecting global DSB levels, we assessed total DSB signals in the *dmc1Δ* background across four chromosomes using pulsed-field gel electrophoresis (PFGE; figure 2*g*,*h*; electronic supplementary material, figure S3). We detected an approximately 25–30% reduction in DSBs in the *dmc1Δ* *spo11-HA* strain relative to *dmc1Δ* (figure 2*g*,*h*). In *rad24Δ* *dmc1Δ*, DSBs were reduced by 10–20% relative to *dmc1Δ* (figure 2*g*,*h*). Finally, in the *dmc1Δ* *rad24Δ* *spo11-HA* strain, DSB frequency fell to just a third of the *dmc1Δ* level (figure 2*g*,*h*). Representative comparisons of lane profile plots are presented in the electronic supplementary material (figure S3). Collectively, these results indicate that the reduced abundance of Spo11-oligo complexes we had observed in *rad24Δ* *dmc1Δ* *spo11-HA* strains (figure 1*b*,*c*) is correlated with a reduced ability to detect DSBs by southern blotting.

In *S. cerevisiae*, as in mammals, meiotic recombination is necessary for accurate chromosome segregation—with errors in this process causing aneuploidy. To determine whether the molecular defects in DSBs and Spo11-oligo abundance correlate with defects in meiotic chromosome segregation, we measured the viability of the haploid spores formed upon completion of meiosis. By itself, the Spo11-HA tag is reported to have very little effect on spore viability [51,53]. We confirmed that *spo11-HA* alone causes no major reduction in spore viability (figure 2*i*). By contrast, in the *rad24Δ* strain, the *spo11-HA* allele caused spore viability to drop from 30% down to less than 5% (figure 2*i*).

### 3.2. Stimulation of double-strand break formation by a Rad24/Mec1(ATR)-dependent positive feedback loop

Mutation of the Rad24-dependent DDR was not expected to affect meiotic DSB formation. The *spo11-HA* allele, on the other hand, is reported to be a DSB formation hypomorph, variably reducing DSBs by 11–50% [51]. Contrary to these expectations, we have made two intriguing observations: (i) in the *DMC1* and *dmc1Δ* backgrounds, DSB frequency at two recombination hotspots (*HIS4::LEU2* and *ARE1*), and across three other chromosomes, is reduced less than we expected by *spo11-HA*; and (ii) DSB frequency, however, is more severely reduced when Rad24 checkpoint activity is abolished in *spo11-HA*; and *spo11-HA dmc1Δ* strains.

Previous analysis of DSB abundance in *spo11-HA* made use of the *rad50S* mutant background [51]. In *rad50S* (as in the similar mutations, *sae2Δ* and *mre11*-‘nuclease-dead’), DSB signals accumulate due to a failure to remove Spo11 from the DSB end [11,20,52]. We remeasured DSB abundance in the *sae2Δ* background at *HIS4::LEU2* (figure 2*j*,*k*)*, ARE1* (see electronic supplementary material, figure S1*c*) and across the length of four chromosomes (figure 2*g*,*h*). In contrast to our previous results in *SAE2*^+^ cells (see figure 2*b*–*h*; electronic supplementary material, figure S1*b*) and now more consistent with the published work [51], we observed DSB signals to be reduced by 50% in the *spo11-HA sae2Δ* strain compared with *sae2Δ* (see figure 2*g*,*h,j*,*k*; electronic supplementary material, figure S1*c*). Moreover, removal of checkpoint activity (*rad24Δ*) in *sae2Δ* strains had little impact on DSB formation in either *SPO11*^+^ or *spo11-HA* (see figure 2*g*,*h,j*,*k*; electronic supplementary material, figure S1*c*).

Taken together, these observations suggest that while *spo11-HA* is deficient in DSB formation (as observed in *sae2Δ*), such a defect is substantially corrected in wild-type and *dmc1Δ* cells by a process that is dependent on the activity of the Rad24 (Mec1/ATR) checkpoint. We propose that these observations reveal the presence of a DDR-dependent positive feedback mechanism that is activated by ssDNA and promotes DSB formation under conditions of suboptimal catalysis (i.e. the *spo11-HA* hypomorph). Consistent with this idea, we note that the final frequency of DSBs that accumulates in *rad24Δ* *dmc1Δ* *spo11-HA* cells (as assessed by PFGE) is very similar to that observed in *sae2Δ* *spo11-HA* (figure 2 *g*,*h*), which, because of the absence of ssDNA intermediates, we expect will also lack efficient Rad24/Mec1 activation [34].

**Figure 3.**
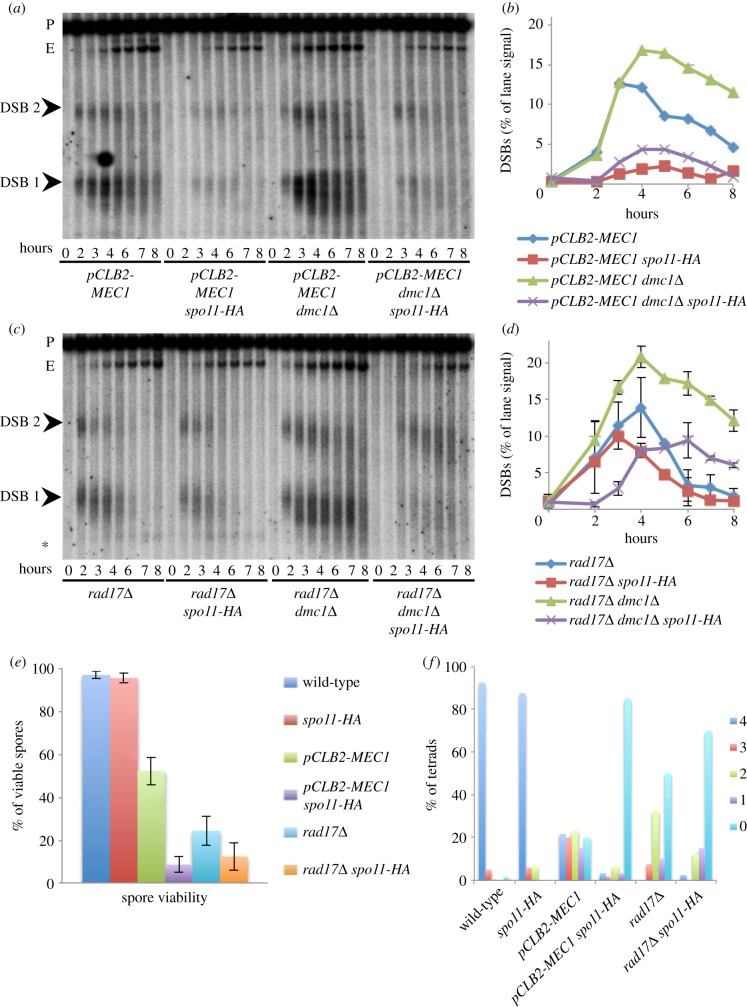
DSB levels are synergistically reduced in *spo11-HA pCLB2-MEC1* and *rad17Δ* mutants. (*a*,*c*) Genomic DNA was isolated at the indicated time points from synchronous cultures of the indicated strains, digested with *Pst*I, fractionated on a 0.7% agarose gel, transferred to nylon membrane and hybridized with the *MXR2* probe. Arrowheads indicate DSB signals; asterisk marks non-specific band; P, parental band; E, ectopic band (see electronic supplementary material, figure S2). (*b*,*d*) Quantification of the total DSB signal (DSB 1+DSB 2) shown in (*a*,*c*) plotted as a percentage of total lane signal. (*e*,*f*) Spore viability for the indicated strains displayed as (*e*) total viability or (*f*) separated according to number of viable spores per tetrad. Error bars in (*e*) are 95% confidence limits.

To determine whether the proposed feedback mechanism was a general feature of the DDR, we repeated experiments in strains mutated for components of either the checkpoint clamp (Rad17) or the Mec1(ATR) kinase itself (figure 3). Owing to problems with synchronizing *mec1* cultures, we used an allele of *MEC1* where expression is specifically repressed during meiosis (*pCLB2-MEC1*; electronic supplementary material, figure S4). DSBs accumulated to high levels in both *rad17Δ* *dmc1Δ* and *pCLB2-MEC1 dmc1Δ* (approx. 16–20%; figure 3*a*–*d*). By contrast, in *spo11-HA* derivatives, DSB levels were reduced to approximately 30% of the *SPO11*^+^ levels, as were ectopic recombination products (see electronic supplementary material, figure S2*d*,*e*), largely mimicking the synergistic DSB defects we observed with *rad24Δ* (figure 2). Furthermore, similar to the synergistic reduction in spore viability observed in *rad24Δ* *spo11-HA* strains (figure 2*i*), loss of the *MEC1* or *RAD17* activity in the *spo11-HA* background also caused a defect in spore viability (figure 3*e*,*f*). Overall, the effects conferred by the loss of Rad17 were less severe than Rad24 or Mec1, suggesting that there may be previously uncharacterized differences between the roles of these DDR components. Nevertheless, we tentatively conclude that the proposed positive feedback mechanism uses all major components of the canonical ssDNA-dependent DDR pathway.

### 3.3. The requirement for positive stimulation of double-strand break formation by Rad24/Mec1 is not unique to the *spo11-HA* hypomorph

The precise reason for the hypomorphic behaviour of the Spo11-HA protein is unknown. To determine whether the positive feedback mechanism we have uncovered is a general feature of meiotic recombination regulation (rather than uniquely required to rescue the *spo11-HA* allele), we investigated whether other Spo11 hypomorphs also benefit from Rad24/Mec1 checkpoint activation. We selected a weak hypomorphic allele of *SPO11*, *spo11-D290A*, which contains a point mutation in the putative Toprim domain thought to aid coordination of a metal ion necessary for DSB catalysis [53]. Under normal conditions, this allele is reported to have extremely mild defects in recombination, but suffer a severe defect at lower temperatures, or when HA-tagged [51,53]. For these reasons, we considered the *spo11-D290A* (untagged) allele to be a good candidate for testing the requirement for checkpoint activity.

**Figure 4.**
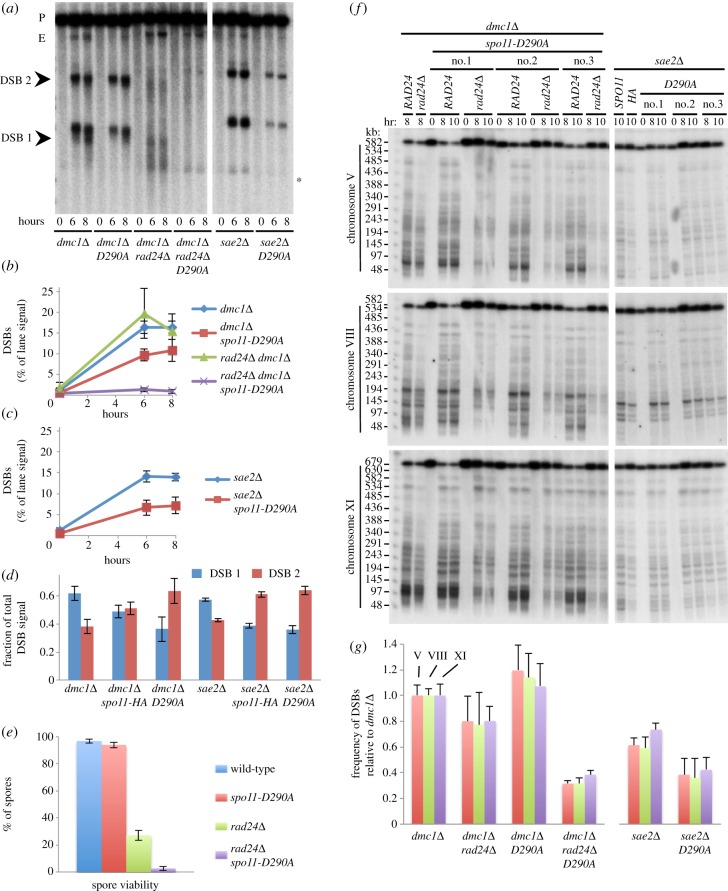
Synergistic reduction in meiotic DSBs in *rad24Δ* *spo11-D290A* strains. (*a*) Genomic DNA was isolated at the indicated time points from synchronous cultures of the indicated strains, digested with *Pst*I, fractionated on a 0.7% agarose gel, transferred to nylon membrane and hybridized with the *MXR2* probe. Arrowheads indicate DSB signals; asterisk indicates non-specific band; P, parental band; E, ectopic recombinant. (*b*,*c*) Quantification of the total DSB signal (DSB 1 + DSB 2) shown in (*a*) plotted as a percentage of total lane signal. (*d*) Proportion of DSB signal present in DSB 1 and DSB 2 for the indicated strains. (*e*) Spore viability displayed as total viability for the indicated strains. Error bars are 95% confidence limits. (*f*) Intact chromosomal DNA was isolated at the indicated time points from synchronously sporulating meiotic cultures, separated by PFGE, transferred to a nylon membrane and sequentially hybridized using a radiolabelled fragment of a gene close to the left telomere of each chromosome (chr V = *RMD6*; chr VIII = *CBP2*; chr XI = *JEN1*). Presented gel shows experiment in triplicate. Intact, full-length chromosomes migrate near the top of the gel. DSBs signals appear as the shorter, faster-migrating bands/molecules present in the 8–10 h time points. (*g*) Quantification of total DSB signals in each lane expressed as a fraction of total lane signal. Measured signals were adjusted to account for the likelihood of multiple DSBs occurring on the same molecule (see material and methods). Because the absolute frequency of DSB formation varies with chromosome, calculated average frequencies (±s.d.) are plotted relative to *dmc1Δ* to aid easier strain-to-strain comparison.

We first measured DSB formation at the *HIS4*::*LEU2* locus (figure 4*a*–*d*). In the *dmc1Δ* background, the *spo11-D290A* allele resulted in a 30% reduction in DSB formation, and a dramatic redistribution of events away from DSB 1 and towards DSB 2 (figure 4*a*,*b*,*d*). These effects were slightly more pronounced than the defects caused by *spo11-HA* (figure 2*b*–*f*), but nevertheless, in the *DMC1*^+^ background, *spo11-D290A* supported almost wild-type levels of spore viability (figure 4*e*). In the *sae2Δ* background, *spo11-D290A* caused a more severe 50% reduction in DSB signal at *HIS4::LEU2* compared with *SPO11* *sae2Δ* (figure 4*a*,*c*), and the same redistribution of events towards DSB 2 (figure 4*d*).

We next estimated DSB formation globally using PFGE and three chromosome probes (figure 4*f*,*g*). We detected no reduction in DSB formation in the *dmc1Δ* *spo11-D290A* background compared with *dmc1Δ* (on average it was 10% greater; figure 4*g*). By contrast, the *spo11-D290A* allele reduced DSB formation in the *sae2Δ* background by approximately 40% (figure 4*f*,*g*), similar to what we had observed for *spo11-HA* (figure 2*h*). Loss of *rad24Δ* checkpoint activity caused DSB levels at *HIS4::LEU2* to drop below our detection limit in the *spo11-D290A dmc1Δ* background (figure 4*a*,*b*), and a 60–70% reduction in DSBs relative to the *dmc1Δ* control, as measured by PFGE (figure 4*f*,*g*), down to the same frequency of DSBs observed in *sae2Δ* *spo11-D290A*. Finally, we asked whether spore survival in the *spo11-D290A* strain required Rad24/Mec1 activity (figure 4*e*). Consistent with our observations made with the *spo11-HA* allele, spore viability in *rad24Δ* *spo11-D290A* fell to just 2.7%.

Collectively, these results support the view that activation of the Rad24/Mec1 checkpoint by ssDNA intermediates acts as a general mechanism to promote DSB formation when the efficiency of Spo11–DSB formation is compromised.

### 3.4. Involvement of Mek1 kinase in the double-strand break feedback mechanism

**Figure 5.**
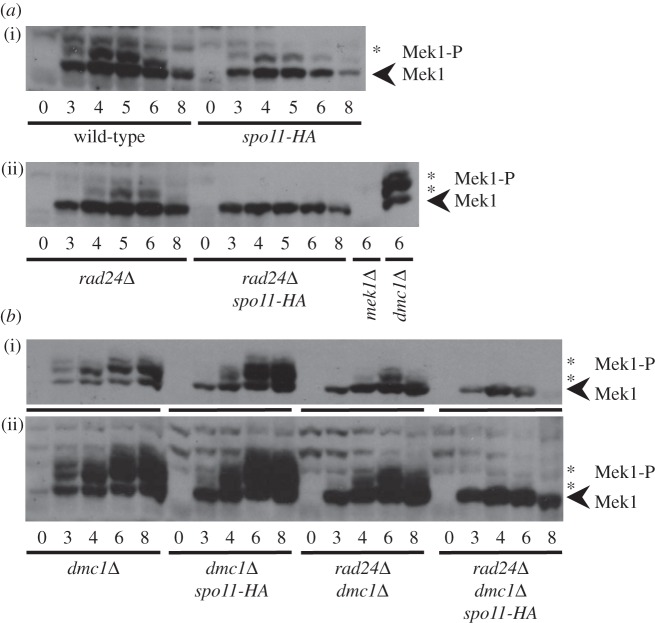
Mek1 phosphorylation is synergistically reduced in a checkpoint-defective *spo11-HA* background. (*a*,*b*) Whole cell lysates from the indicated time points and strains were resolved by SDS-PAGE containing 10 µM phos-tag reagent and probed with anti-Mek1 antibody. Mek1 is indicated with an arrowhead; phosphorylated Mek1 by an asterisk. (*b*) The blot in (ii) is a longer exposure of the blot in (i).

Activation of the DDR kinases (Mec1 and Tel1 in budding yeast) causes phosphorylation of Hop1 and subsequent activation of Mek1 [34], a meiotic kinase with roles related to Rad53/CHK2 [36–39]. Mek1 activation is associated with phosphorylation on a number of residues [35]. To investigate the relationship between Mek1 activity and the proposed feedback process, we assessed the abundance of phosphorylated forms of Mek1 using phos-tag SDS-PAGE and western blotting (figure 5*a*,*b*). In wild-type and *spo11-HA* cultures, slower migrating phosphorylated forms of Mek1 appear coincidently with the time of DSB recombination intermediates (3–6 h; figure 5*a*(i)). Phosphorylated Mek1 was slightly delayed in *rad24Δ* cells, suggesting a partial requirement for Rad24 to achieve Mek1 activation (figure 5*a*(ii)). Strikingly, phosphorylation of Mek1 was completely abolished in the *rad24Δ* *spo11-HA* strain (figure 5*a*(ii)).

Because Mek1 phosphorylation signals are transient in *DMC1*^+^ cells, we repeated the analysis in the *dmc1Δ* background, where DSB recombination intermediates and Mek1 signals would usually be expected to persist (figure 5*b*). In *dmc1Δ* and *dmc1Δ* *spo11-HA*, there was a steady accumulation in the abundance of multiple-shifted species of Mek1, indicating varying levels of phosphorylation (figure 5*b*). In the *rad24Δ* *dmc1Δ* strain, much of this hyper-phosphorylation was lost, confirming that hyper-activation of Mek1 is Rad24-dependent (figure 5*b*). In the *rad24Δ* *spo11-HA dmc1Δ* strain, all residual Mek1 phosphorylation was abolished (figure 5*b*), and not visible even when overexposing the film (figure 5*b*(ii)). Similar loss of Mek1 phosphorylation was observed in *pCLB2-MEC1 dmc1Δ* *spo11-HA* strains (data not shown). Thus, activated Mek1 correlates strongly with the ability to accumulate appreciable levels of meiotic DSBs.

### 3.5. Reduction of double-strand breaks in *spo11-HA rad24Δ* cells is not due to altered repair partner choice

**Figure 6.**
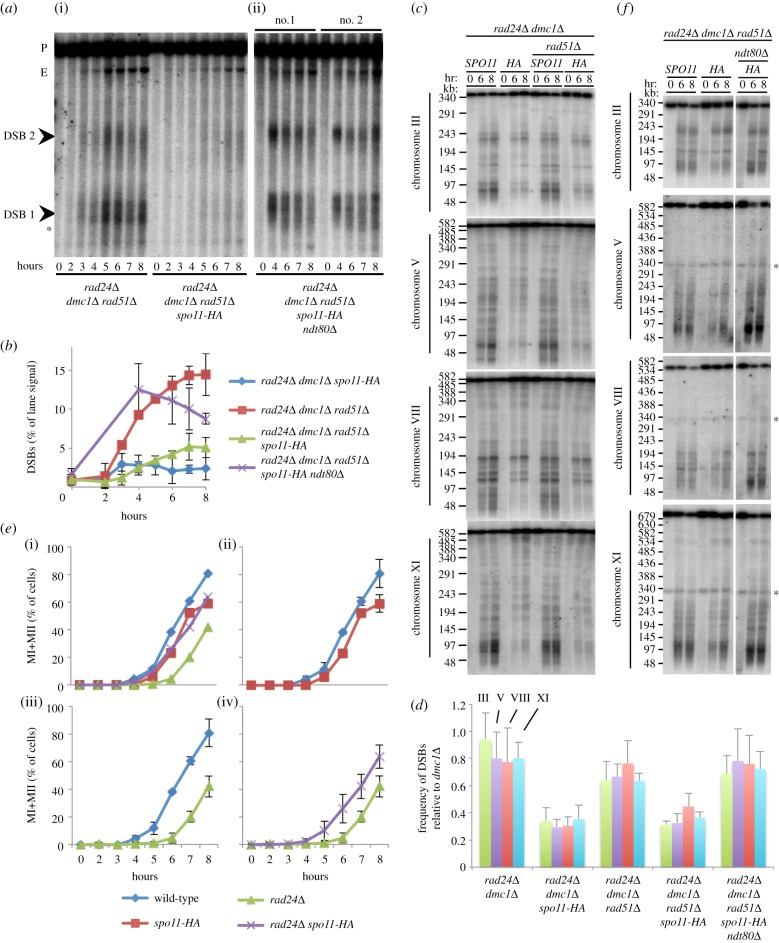
Ndt80-dependent meiotic progression and not inter-sister repair is responsible for the decreased DSB signal in *rad24Δ* *spo11-HA*. (*a*) Genomic DNA was isolated at the indicated time points from synchronous cultures of the indicated strains, digested with *Pst*I, fractionated on a 0.7% agarose gel, transferred to nylon membrane and hybridized with the *MXR2* probe. Arrowheads indicate DSB signals, asterisk marks non-specific band; P, parental band, E, ectopic band. (*b*) Quantification of the total DSB signal (DSB 1 + DSB 2) shown in (*a*) plotted as a percentage of total lane signal. *rad24Δ* *dmc1Δ* *spo11-HA* data from figure 2*f* are overlaid to aid visual comparison. (*c*,*f*) Intact chromosomal DNA was isolated at the indicated time points from synchronously sporulating meiotic cultures, separated by PFGE, transferred to a nylon membrane and sequentially hybridized using a radiolabelled fragment of a gene close to the left telomere of each chromosome (chr III = *CHA1*; chr V = *RMD6*; chr VIII = *CBP2*; chr XI = *JEN1*). Intact, full-length chromosomes migrate near the top of the gel. DSBs signals appear as the shorter, faster-migrating bands/molecules present in the 6–8 h time points. Asterisk marks residual parental signal from the chromosome III hybridization. (*d*) Quantification of total DSB signals in each lane expressed as a fraction of total lane signal. Measured signals were adjusted to account for the likelihood of multiple DSBs occurring on the same molecule (see material and methods). Because the absolute frequency of DSB formation varies with chromosome, calculated average frequencies (±s.d.) are plotted relative to *dmc1Δ* to aid easier strain-to-strain comparison. (*e*) Samples were stained with DAPI and the average fraction of cells having undergone the first and/or second meiotic division was plotted ±s.d. (*n* > 200). (i) Shown without error bars for clarity. (ii–iv) Individual comparisons.

During meiosis, DSB repair reactions take place preferentially between homologous chromosomes rather than between sister chromatids [54,55]. The Mec1/Tel1 DDR checkpoint machinery is important for enforcing such ‘repair partner choice’ via activation of the Hop1 adapter protein and the Mek1 kinase [34,44]. It seemed plausible that the synergistic loss of DSB and Spo11-oligo signals, and drop in spore viability caused by the combination of *spo11-HA* and *rad24Δ*, could be explained by the observed failure to fully activate Mek1, and thus to appropriately control repair partner choice, resulting in increased occurrence of Rad51-dependent inter-sister recombination. To test this idea, we repeated our DSB analyses in *rad51Δ* *dmc1Δ* strains, thereby preventing all recombination-mediated pathways of DSB repair (figure 6). As expected, in the *rad24Δ* *rad51Δ* *dmc1Δ* strain, DSB signals at *HIS4::LEU2* accumulated, reaching a plateau of approximately 15% of total lane signal (figure 6*a*,*b*). In the *rad24Δ* *rad51Δ* *dmc1Δ* *spo11-HA* strain, despite having removed the potential for both Rad51 and Dmc1-dependent repair, accumulated DSB signals were barely increased relative to *rad24Δ* *dmc1Δ* *spo11-HA*, remaining approximately threefold lower than the *SPO11*^+^ (figure 6*a*,*b*) control. Analysis of DSB formation across four chromosomes revealed a similar relationship (figure 6*c*,*d*). Specifically, we detected no increase in DSB abundance in *rad24Δ* *dmc1Δ* *rad51Δ* *spo11-HA* relative to *rad24Δ* *dmc1Δ* *spo11-HA* (see figure 6*c*,*d*; electronic supplementary material, figure S3). We conclude that the loss of DSB signal observed in *rad24Δ* *spo11-HA* strains is not primarily due to changes in repair partner choice.

### 3.6. Reduction of double-strand break formation in *spo11-HA rad24Δ* cells is due to precocious Ndt80 activity

DDR mutants initiate the first meiotic nuclear division about 1 h later than wild-type cells [40] (figure 6*e*). This delay is thought to be caused by errors in recombination. We noted that the *spo11-HA* allele alleviated the meiotic delay observed in the *rad24Δ* strain (figure 6*e*), consistent with the observed synergistic reduction in DSB signal alleviating recombination problems. However, it was possible that the reduced DSB signal in *rad24Δ* *spo11-HA* and *rad24Δ* *dmc1Δ* *spo11-HA* cells compared with *rad24Δ* *SPO11*^+^ cells was itself due to the more rapid cell cycle progression beyond prophase I that arises in the *rad24Δ* *spo11-HA* double mutant compared with *rad24Δ* *SPO11*^+^ (figure 6*e*).

Exit from prophase I into anaphase I is controlled by expression of the Ndt80 transcription factor [56,57]. To distinguish between these two possibilities, DSB abundance was assessed in the *ndt80Δ* *rad24Δ* *rad51Δ* *dmc1Δ* *spo11-HA* background, where all DSB signals should accumulate, and where the cells will be forced to arrest at late prophase I in a checkpoint-independent manner owing to deletion of the *NDT80* gene. To our surprise, the *ndt80Δ*-enforced prophase arrest was sufficient to restore DSB accumulation at *HIS4*::*LEU2* to the otherwise DSB-deficient *rad24Δ* *rad51Δ* *dmc1Δ* *spo11-HA* strain (figure 6*a*(ii)).

To confirm that the restoration of DSB formation by *ndt80Δ*-enforced arrest was not unique to *HIS4::LEU2*, we measured DSB formation across four chromosomes using PFGE (figure 6*d*,*f*). Consistent with our results at *HIS4::LEU2*, the *ndt80Δ* arrest caused DSB signals to return to levels indistinguishable from the *rad24Δ* *dmc1Δ* *rad51Δ* control (figure 6*d*,*f*). Taken together, our results suggest that the apparent positive feedback on meiotic DSB formation is mediated by Mec1-dependent inhibition of Ndt80.

### 3.7. Transient *ndt80*-induced prophase arrest improves spore viability in hypomorphic *spo11 rad24Δ* strains

Our results suggest that the synergistic reduction in DSB signal observed in *rad24Δ* *spo11-HA* and *rad24Δ* *spo11-D290A* strains is due to precocious Ndt80 expression and/or activation causing more rapid passage through meiotic prophase, which we suppose prevents efficient DSB formation by the hypomorphic Spo11-HA and Spo11-D290A proteins.

**Figure 7.**
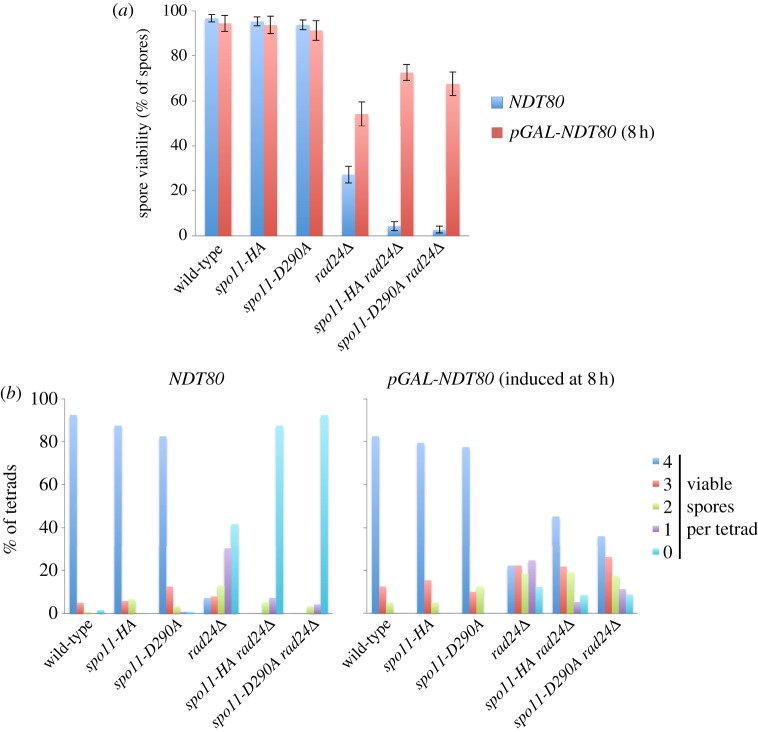
Transient prophase arrest rescues *rad24Δ* spore viability defects. (*a*,*b*) Spore viability displayed as (*a*) total viability or (*b*) separated according to number of viable spores per tetrad for the indicated strains. Error bars are 95% confidence limits. *NDT80* indicates spore viability of normally sporulated strains. *pGAL-NDT80* derivatives were transiently arrested in prophase until 8 h, then induced to sporulate by addition of beta-oestradiol to 2 µM.

If this idea is correct, we reasoned that transiently extending meiotic prophase might be sufficient to restore DSB formation (and the subsequent crossover formation necessary for accurate chromosome segregation) to checkpoint-defective Spo11 hypomorphs. To test this idea, we placed the *NDT80* transcription factor under the control of the *GAL1-10* promoter, where expression can be induced by addition of oestradiol to the growth media [58]. As expected, without addition of oestradiol, cells failed to sporulate, arresting permanently in meiotic prophase, similar to what occurs upon deletion of the *NDT80* gene (data not shown). By contrast, samples induced with oestradiol after 8 h in meiosis sporulated efficiently and permitted us to assess the viability of the haploid spores (figure 7). Dramatically, we observed spore viability in both the *rad24Δ* *spo11-HA* and *rad24Δ* *spo11-D290A* strains to reach 73% and 68%, respectively—increases of approximately 20-fold—as a consequence of simply extending the period in meiotic prophase (figure 7). Intriguingly, the transient *ndt80*-arrest improved spore viability significantly above that of the *rad24Δ* strain (figure 7). This suggests that one of the reasons for low spore viability in *rad24Δ* cells is due to untimely onset of the meiotic divisions—perhaps before all DSB repair has finished. Collectively, these results provide compelling evidence for the activation of the Rad24/Mec1 checkpoint pathway being critical for the orchestration of meiotic recombination events with the meiotic nuclear divisions.

### 3.8. Cdc5 depletion does not permit double-strand break formation in *rad24Δ* *dmc1Δ* *spo11-HA* strains

To determine whether the DSB defects in *rad24Δ* strains is directly due to Ndt80 expression or instead to the onset of anaphase I, which ensues as a result of the Ndt80-dependent transcriptional cascade, we asked whether arresting cells at the metaphase–anaphase transition is sufficient to restore DSB formation in *NDT80*^*+*^ *rad24Δ* *dmc1Δ* *spo11-HA* cells. The budding yeast Polo-like kinase, Cdc5, regulates late prophase events including Holliday junction resolution and the dissociation of Rec8 cohesin from meiotic chromosomes to enable their segregation [59,60]. Because *CDC5* is an essential gene, we took advantage of a *pCLB2-CDC5* allele developed previously, which, similar to our *pCLB2*-*MEC1* allele, is not expressed during meiosis [59,60].

Depletion of Cdc5 caused cells to arrest in prophase (data not shown), yet had little effect on measured DSB frequency at *HIS4::LEU2* (see electronic supplementary material, figure S5). In *rad24Δ* *dmc1Δ* *pCLB2-CDC5* cells, DSB frequencies rose to 25% of total DNA at late time points (see electronic supplementary material, figure S5), a modest increase relative to *CDC5*^+^ that may be due to prolonged prophase I arrest. Cdc5 depletion also had almost no effect on DSB accumulation in the *rad24Δ* *dmc1Δ* *spo11-HA* background, with measured signals barely rising above general lane background (see electronic supplementary material, figure S5). Our observation that DSB formation is restored by *NDT80* deletion (figure 6), but not by Cdc5 depletion, points to a direct inhibitory role of Ndt80 itself, or of a protein other than Cdc5 that is upregulated by Ndt80 expression, rather than an indirect consequence of prophase arrest.

## 4. Discussion

### 4.1. A model for regulation of double-strand break formation

Meiotic DSB formation is regulated at numerous levels by relatively poorly understood mechanisms. Although Spo11 is the evolutionarily conserved catalytic component, its activity depends on interactions made between itself and the accessory DSB complex proteins, and with the chromosomal substrate. Spo11 activity is modulated temporally by the local timing of DNA replication [61] and by cell cycle-dependent phosphorylation events, such as phosphorylation of Mer2 by cyclin-dependent kinase (CDK) and Dbf4-dependent kinase (DDK) [62–64], and spatially by histone occupancy [48] and their post-translational modification [65,66], and by meiosis-specific chromosome structure components [48,67].

**Figure 8.**
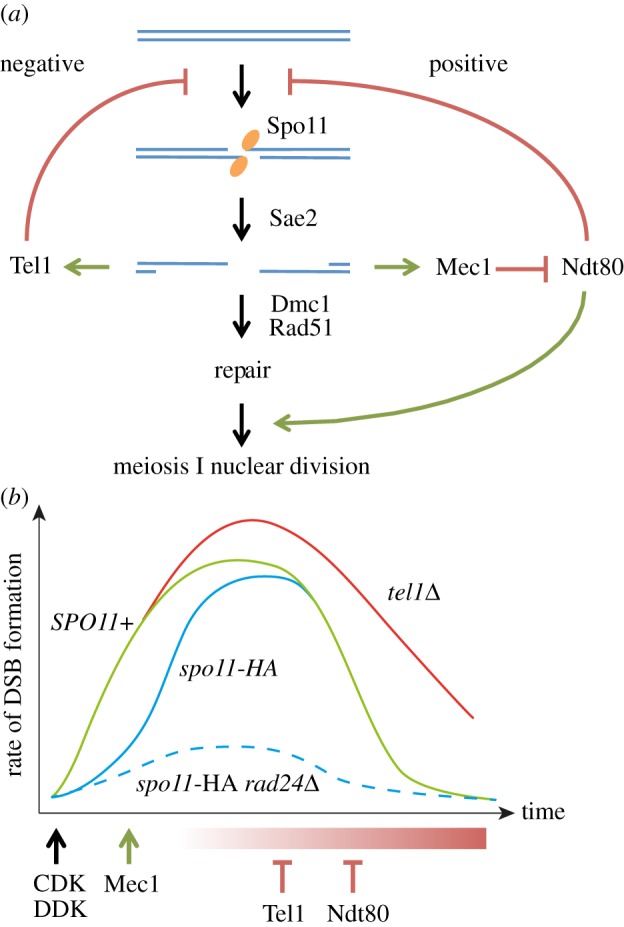
Model for checkpoint-dependent regulation of DSB formation. (*a*) DSB formation triggers Mec1/Tel1-dependent positive and negative feedback loops, which modulate the rate of DSB formation. The Rad24/Mec1-dependent positive stimulation of DSB formation is activated by ssDNA formation, leading to transient Ndt80 inactivation, and is required to overcome defects in DSB formation (i.e. *spo11-HA* and *spo11-D290A* backgrounds). Negative regulation of DSB formation is mediated by both Tel1 and Ndt80. (*b*) Cartoon to describe the relative changes in rate of DSB formation over time in meiotic prophase for various mutants (see text for details).

Here, by using an allele of Spo11, which is hypomorphic for DSB catalysis, we additionally reveal that DSB formation is modulated in a positive manner by activation of the DNA damage checkpoint machinery Mec1(ATR). Recent work in mice, flies and yeast [45–47], and additional observations from our own laboratory, suggest that the complementary Tel1(ATM) kinase pathway acts to inhibit DSB formation (S.G., R.A., V.G. & M.J.N. 2012, unpublished data). Collectively, these results point to the interaction of two regulatory loops that appear to work antagonistically to modulate DSB frequency, thereby creating a moderate level of recombination that is neither too high nor too low (figure 8*a*).

Our model contains a number of features (figure 8*a*,*b*): the rate of Spo11–DSB formation is initially increased concomitantly with CDK/DDK-dependent phosphorylation of Mer2 [62–64], but reaches a plateau that we suggest is at least in part due to the activation of Tel1 and Ndt80 (see below). We propose that in the hypomorphic *spo11-HA* and *spo11-D290A* strains, DSB accumulation is initially slower, but that activation of the Rad24/Mec1(ATR) kinase by ssDNA formation at emergent DSBs causes transient inhibition of Ndt80, which enables the rate of DSB formation to increase until it reaches a plateau similar to that observed in wild-type cells. Therefore, activation of this positive feedback results in the similar efficiency of DSB formation that we observe in *SPO11*^+^ relative to *spo11-HA* or *spo11-D290A* strains. By contrast, in the absence of the Mec1 checkpoint pathway, precocious Ndt80 activity results in a residual low rate of DSB formation in the hypomorphic *spo11* strains. Our model is consistent with the increased Ndt80 transcription observed in the *dmc1Δ* *rad17Δ* checkpoint mutant relative to *dmc1Δ* [68]. We suggest that a comparable effect is observed in *sae2Δ* *spo11-HA* and *sae2Δ* *spo11-D290A* strains, which, owing to an inability to produce ssDNA, may also exhibit precocious Ndt80 activation.

Overall, our results are surprising given the proposed negative role of ATM in DSB formation in mouse and flies [45,46], and the negative role in DSB formation reported at the *HIS4::LEU2* hotspot for Tel1 in *S. cerevisiae* [47]. We assume that over time Tel1 becomes hyper-activated by the presence of multiple ongoing recombination intermediates, and that this then downregulates DSB formation. As such, mutation of *TEL1* increases the rate and/or prolongs the activity of the Spo11–DSB machinery (S.G., R.A., V.G. & M.J.N. 2012, unpublished data).

It is worth noting that we have revealed the requirement of the Mec1 checkpoint response in situations where Spo11 activity is downregulated due to defined genetic modification. Although we are unable to exclude that the mechanism we describe reflects a unique consequence of this mutant situation, we interpret our results to indicate that the transient prophase delay caused by Mec1 activation will also promote meiotic DSB formation in other suboptimal circumstances. For example, mutation in a gene other than Spo11, defects in chromosome pairing and/or synapsis or perhaps slower Spo11–DSB formation for an environmental reason (see below).

### 4.2. Possible mechanisms of regulation

Mec1 and Tel1 are members of the PIKK-family, and preferentially phosphorylate substrate serine and threonine residues that precede a glutamine (SQ/TQ sites [69]). One of the many intriguing points of our study is how the two kinases are able to elicit both positive and negative effects. This could be explained if the rate of phosphorylating different substrates changes with kinase activity. For example, an initial low-level phosphorylation of an abundant (and easily phosphorylatable) target might promote DSB formation, but upon hyper-activation of Mec1/Tel1, additional substrates become phosphorylated that begin to negatively impact on DSB formation. Candidate targets are many, and could be histones, chromosomal structural proteins or members of the DSB-forming complex itself. The chromosomal protein, Hop1—a known target of Mec1 and Tel1 [34]—is involved in both DSB formation and DSB repair, and more recently Rec114 (a member of the Spo11–DSB complex) has been identified as a target of Mec1/Tel1 [70]. Our results also implicate the involvement of the downstream kinase, Mek1—whose substrates currently remain unclear—and of Ndt80, the transcription factor required to promote exit from meiotic prophase. Defining the relevant targets of Ndt80 and these three kinases, and of their mechanism of action, are important future goals if we are to fully understand how DSB formation is regulated.

The spatial and temporal distributions of meiotic DSB formation may also be modulated by meiotic chromosome morphogenesis. For example, chromosome axis-associated proteins involved in Spo11–DSB formation are excluded from synapsed chromosomes [71,72], suggesting that synapsis itself may promote their eviction. Consistent with this idea, in mouse spermatocytes, regions of chromosomes that fail to synapse display increased density of DSB markers [73]. Therefore, it remains possible that under conditions of suboptimal Spo11–DSB formation, concomitant reductions in chromosome pairing and synapsis will cause an increase in DSB formation at chromosomal regions that have yet to become paired or synapsed [70,73]. Such a homeostatic mechanism, which could arise via a reduction of *cis-* and/or *trans-*inhibition, is similar to that discussed by Zhang *et al.* [47]. We wish to emphasize that the transient prophase arrest activated by the Mec1(ATR)- or Tel1(ATM)-dependent damage response (as we observe in this work) could ensure that progression into late stages of meiotic prophase does not occur before sufficient numbers of recombination interactions have been established to ensure accurate meiotic chromosome segregation.

### 4.3. Differential effects on double-strand break formation across the *HIS4::LEU2* locus

The *HIS4::LEU2* DSB site 1 hotspot arose fortuitously via the ectopic insertion of a short bacterial DNA sequence during integration of the *LEU2* marker downstream of the *HIS4* gene [74]. DSB site 1 remains one of the strongest recombination sites characterized in the budding yeast genome [48]. The precise reason for becoming such a strong DSB site is not known, but if we assume DSB site 1 conforms to the general mechanisms regulating DSB formation at natural sites, it is likely to be due to the creation of a higher-order chromatin structure that makes the local DNA sequence particularly receptive to Spo11–DSB activity [48]. Given this idea, it is intriguing that the two Spo11 hypomorphs, *spo11-HA* and *spo11-D290A*, have such a severe effect on DSB site 1, but not at site 2 nor at DSB formation generally across the genome.

DSB site 1 is particularly narrow relative to its heat (see electronic supplementary material, figure S6). When compared to all other mapped DSB hotspots [48], DSB site 1 is narrower than 60% of all hotspots (see electronic supplementary material, figure S6*a*), yet is in the top 3% of sites ranked according to the frequency of mapped Spo11-oligonucleotides (see electronic supplementary material, figure S6*b*)—with Spo11-oligo hits about 10 times greater than at other similarly narrow sites. Together, this makes DSB site 1 have the second highest density of mapped Spo11-oligonucleotides per bp (see electronic supplementary material, figure S6*c*), and a significant outlier relative to hotspots of similar width (see electronic supplementary material, figure S6*a*). We speculate that these unique characteristics render DSB site 1 particularly sensitive to reductions in Spo11's catalytic activity. By contrast, DSB site 2 maps to the promoter of the inserted *LEU2* gene, and probably behaves in a way more similar to canonical DSB sites and provides a buffer for defects in DSB formation at site 1.

DSB site preference was also modified by loss of Rad24 activity. However, in this case, *rad24Δ* caused an increase in the frequency of DSBs at site 1 (figure 2). Although we do not know the precise reason for this, one explanation is that while Rad24/Mec1 acts generally to promote DSB formation via inactivation of Ndt80, Rad24/Mec1 may also act locally to inhibit DSB formation at the equivalent locus on the sister chromatid or homologous chromosome [47]. The fact that such effects are observed in *dmc1Δ* recombination mutants—which are thought to lack DNA strand exchange interactions between homologous chromosomes—suggests that such Rad24-dependent *trans*-inhibition may predominantly take place between sister chromatids.

### 4.4. Double-strand break homeostasis versus crossover homeostasis

During meiosis, only a portion of DSBs repair as interhomologue crossover events—the prerequisites for chiasma formation and the accurate segregation of homologous chromosomes. In budding yeast, about half of total DSB events resolve as a crossover [49,75], whereas in mammals the fraction is less than one-tenth [76]. An excess of precursor events (DSBs) may provide a large buffering pool from which to generate the essential, but smaller, number of crossovers. Such a system has the potential to better tolerate cell-to-cell variation in DSB number. The name used to describe this phenomenon is crossover homeostasis, and it was revealed via the analysis of a hypomorphic series of Spo11 alleles that systematically reduce DSB frequency [51]. Martini *et al.* uncovered a nonlinear relationship between gene conversion and crossing over at a modified *ARG4* locus, supporting the notion of crossover homeostasis [51]. However, it was not reported whether the hypomorphic *SPO11* series affected DSB frequency and/or DSB distribution at this locus (such as we have witnessed at *HIS4::LEU2*). Changes in either parameter can alter measured gene conversion frequencies, and chromosomal estimates of DSB formation were made in a *rad50S* background, where, like *sae2Δ*, Spo11–DSBs accumulate without ssDNA resection [52].

Our work here suggests that the measured frequency of DSB formation obtained in such hypomorphic Spo11 strains may be substantially different in *rad50S*/*sae2Δ* when compared with resection-proficient wild-type or *dmc1Δ* strains due to the activation of Mec1 substantially improving DSB formation. Assuming our observations are an accurate reflection of DSB formation in repair-proficient (*DMC1*^+^) meiosis, then the strength of crossover homeostasis may need to be reviewed. Alternatively, it is possible that the substantial increases in DSB formation we report for *dmc1Δ* *spo11-HA* and *dmc1Δ* *spo11-D290A* compared with *sae2Δ* *spo11-HA* and *sae2Δ* *spo11-D290A* are themselves overestimates due to checkpoint hyper-activation stimulating DSB formation in a manner that would not occur in repair-proficient cells. Developing accurate and quantitative measures of genome-wide recombination is needed to resolve such conundrums.

### 4.5. Why did checkpoint-dependent regulation of double-strand break formation arise?

The initiation of meiotic recombination by DSB formation is a potentially catastrophic event for the maintenance of genome stability: any unrepaired DSB can result in the loss of genetic information upon nuclear division. In this context, it makes sense for the DSB programme to be tightly temporally regulated: DSBs should form only in meiosis, and only after local DNA replication has occurred. DSB formation and repair must also occur efficiently across the genome prior to the onset of anaphase. We suggest that the positive and negative action of the meiotic checkpoint helps to make this system efficient and robust under what may often be suboptimal conditions. At this point, we note that budding yeast did not evolve to enter meiosis under the controlled laboratory conditions in which most experiments are performed. In the wild, variation in genotype and in the local environment may cause substantial deficiencies in these processes. Indeed, effects on recombination caused by polymorphism, temperature and nutritional deficiencies have all been reported [49,77–79]. In this context, activation and utilization of the DNA damage response may be just one of many redundant mechanisms that help to modulate appropriate recombination outcome.

## 5. Material and methods

### 5.1. Yeast strains and culture methods

Meiotic cultures were prepared as follows: YPD cultures (1% yeast extract/2% peptone/2% glucose) were diluted 100-fold into YPA (1% yeast extract/2% peptone/1% K-acetate) and grown vigorously for 14 h at 30°C. Cells were collected by centrifugation, washed once in water, resuspended in an equal volume of prewarmed 2% K-acetate containing diluted amino acid supplements and shaken vigorously at 30°C. *Saccharomyces cerevisiae* strains (see electronic supplementary material, table S1) are isogenic to the SK1 subtype and were generated using standard genetic techniques. The base strain genotype is *ho::LYS2*, *lys2*, *ura3*, *arg4-nsp*, *leu2::hisG*, *his4X::LEU2*, *nuc1::LEU2*. Spo11 protein is tagged with the HA3-His6::*kanMX4* epitope [53]. The *spo11-D290A::kanMX4* allele is a single copy point mutation integrated without epitope tag at the natural Spo11 locus [53]. *dmc1Δ*::*LEU2*, *dmc1Δ*::*hphMX*, *sae2Δ*::*kanMX6*, *mek1Δ*::*URA3*, *rad51Δ*::*hisG-URA3-hisG*, *rad17Δ*::*natMX*, *rad24Δ*::*hphMX* and *ndt80Δ*::*hphMX* are all full replacements of the ORF with the selection marker. In the *pCLB2-MEC1* and *pCLB2*-*CDC5* alleles, the natural *MEC1* or *CDC5* promoter is replaced with the *CLB2* promoter, whose activity is downregulated upon meiotic entry (see electronic supplementary material, figure S4 [59,60,80,81]). The *pGAL-NDT80::TRP1* strains replace the *NDT80* promoter with the *GAL1-10* promoter sequence and include the *GAL4::ER* chimeric transactivator for oestradiol-induced expression [58].

### 5.2. Double-strand break analysis

DSB signals were detected using standard techniques by indirect end-labelling of specific genomic loci after fractionation and transfer to nylon membranes [82]. For DSBs at *HIS4:LEU2*, genomic DNA was digested with *Pst*I, fractionated on 0.7% agarose in 1× tris-acetate-EDTA (TAE) for approximately 18 h at room temperature, transferred to nylon membrane under denaturing conditions then hybridized with a probe that recognizes the *MXR2* locus. For DSBs at *ARE1*, genomic DNA was digested with *Bgl*II, fractionated and transferred as above, and then hybridized with a probe that recognizes the *RSC6* locus. To measure the total DSB formation along whole chromosomes, chromosomal-length DNA was prepared after first immobilizing cells in agarose plugs as described [82]. Chromosomes were fractionated using a CHEF-DRIII PFGE system (BioRad) using the following conditions: 1.3% agarose in 0.5× TBE; 14°C; 6 V cm^–1^; switch angle 120°; figures 2*g* and 6*c*, ramped switch time of 15–22 s over 25 h, then constant switch time of 45 s for 4 h; figures 4*f* and 6*f*, ramped switch time of 20–50 s over 28 h. After transfer to nylon membrane under denaturing conditions, genomic DNA was sequentially hybridized with DNA probes that localize close to the left telomere of four chromosomes. Probes used were *CHA1* (Chr III)*, RMD6* (Chr V), *CBP2* (Chr VIII) and *JEN1* (Chr XI). Between each hybridization, radiolabelled probes were removed by sequential washes in 0.4 M NaOH (2 × 5 min), 2× SSC (2 × 5 min) and six rinses in distilled water, and then air-dried on Whatman paper for 1 h and stored at 4°C. Radioactive signals were collected on phosphor screens, scanned with a Fuji FLA5100 and quantified using ImageGauge software (FujiFilm). DSBs at *HIS4::LEU2* are reported as a percentage of the total lane signal after background subtraction. For analysis of PFGE experiments, DSBs occurring far from the probed chromosome end will be underrepresented whenever they arise on a chromosome with additional DSBs closer to the probe. To correct for this, the measured frequency of DSBs (fraction of total lane signal) was estimated using the following standard formula: corrected DSB fraction = –ln(1 – measured DSB fraction). This correction assumes that DSBs at different loci on the same chromosome occur independently of one another.

Values plotted with standard deviation bars are the mean of at least two independent time-course experiments. For PFGE experiments, all data are the average of at least three independent time courses of each strain except for *rad24Δ* *dmc1Δ* (two experiments). Where the exact same sample has been analysed on different gels, the average measure for that sample is used before calculating the average and standard deviation of the experimental repeats. For PFGE data, DSB frequencies for all strains are expressed relative to the average frequency of DSBs forming in *dmc1Δ* strain.

### 5.3. Meiotic progression

Cells were fixed in 100% methanol and aliquots mixed with 1 µg ml^−1^ DAPI. Cells were scored for the presence of one, two or four nuclei and percentage at each time point calculated.

### 5.4. Spore viability

Fresh diploid colonies were incubated in 2% K-acetate liquid for 48 h at 30°C, and then incubated with zymolyase 100 T at a final concentration of 10 µg ml^−1^ in a 150 mM sodium phosphate buffer at 37°C for 10 min. Dissected spores were incubated for 2 days at 30°C on YPD and scored for percentage viability per strain and viable spores per tetrad. For the *pGAL-NDT80* experiments, synchronized cultures were split after 8 h in 2% K-acetate, and one fraction induced to sporulate by addition of beta-oestradiol to a final concentration of 2 µM. Cultures were then incubated for a further 40 h at 30°C prior to dissection. Where shown, error bars are 95% confidence limits.

### 5.5. Spo11-oligonucleotide assay

Spo11-oligonucleotide complexes were detected by immunoprecipitation and end-labelling following established methods [11,18,50]. Briefly, cells were broken in 10% ice-cold TCA using zirconium beads and a BioSpec 24. Precipitated material was dissolved in SDS buffer, diluted with Triton X100, and Spo11 was immunoprecipitated from total soluble protein using anti-HA antibody (F-7; Santa Cruz Biotechnology) and protein-G-agarose (Roche). Oligonucleotide complexes were 3′-labelled with α-^32^P dCTP using Terminal deoxynucleotidyl transferase (Fermentas) and fractionated on a 7.5% SDS-PAGE gel. Following transfer to polyvinylidene difluoride (PVDF) membrane, radioactive signals were collected on phosphor screens, scanned with a Fuji FLA5100 and quantified using ImageGauge software.

### 5.6. Western blotting

Mek1 was detected from TCA-denatured whole cell lysates dissolved in 2% SDS/0.5 M Tris–HCl pH 8.1/0.005% bromophenol blue. Total soluble protein was fractionated on 7% SDS-PAGE containing 10 µM phos-tag (Wako) and 20 µM MnCl_2_ for 3 h at 100 V. Gels were soaked in 1× CAPS transfer buffer (10 mM CAPS/10% methanol) containing 1 mM EDTA for 10 min and then transferred to PVDF membrane in 1 × CAPS for 1 h at constant 0.6 A. Membrane was probed with anti-Mek1 (1 : 2500; kindly provided by Pedro San-Segundo [35]), followed by anti-Rabbit-HRP (1 : 3000).

## Supplementary Material

Supplementary table and supplementary figures 1-6

## References

[1] Neale MJ Keeney S 2006 Clarifying the mechanics of DNA strand exchange in meiotic recombination. Nature 442, 153–158 (doi:10.1038/nature04885) 16838012 10.1038/nature04885 PMC5607947

[2] Keeney S 2001 Mechanism and control of meiotic recombination initiation. Curr. Top. Dev. Biol. 52, 1–53 (doi:10.1016/S0070-2153(01)52008-6) 11529427 10.1016/s0070-2153(01)52008-6

[3] Sun H Treco D Szostak JW 1991 Extensive 3′-overhanging, single-stranded DNA associated with the meiosis-specific double-strand breaks at the *ARG4* recombination initiation site. Cell 64, 1155–1161 (doi:10.1016/0092-8674(91)90270-9) 2004421 10.1016/0092-8674(91)90270-9

[4] Nairz K Klein F 1997 *mre11S*—a yeast mutation that blocks double-strand-break processing and permits nonhomologous synapsis in meiosis. Genes Dev. 11, 2272–2290 (doi:10.1101/gad.11.17.2272) 9303542 10.1101/gad.11.17.2272 PMC275393

[5] Prinz S Amon A Klein F 1997 Isolation of *COM1*, a new gene required to complete meiotic double-strand break-induced recombination in *Saccharomyces cerevisiae*. Genetics 146, 781–795 9215887 10.1093/genetics/146.3.781 PMC1208051

[6] McKee AH Kleckner N 1997 A general method for identifying recessive diploid-specific mutations in *Saccharomyces cerevisiae*, its application to the isolation of mutants blocked at intermediate stages of meiotic prophase and characterization of a new gene *SAE2*. Genetics 146, 797–816 9215888 10.1093/genetics/146.3.797 PMC1208052

[7] Furuse M Nagase Y Tsubouchi H Murakami-Murofushi K Shibata T Ohta K 1998 Distinct roles of two separable in vitro activities of yeast Mre11 in mitotic and meiotic recombination. EMBO J. 17, 6412–6425 (doi:10.1093/emboj/17.21.6412) 9799249 10.1093/emboj/17.21.6412 PMC1170966

[8] Tsubouchi H Ogawa H 1998 A novel *mre11* mutation impairs processing of double-strand breaks of DNA during both mitosis and meiosis. Mol. Cell Biol. 18, 260–268 9418873 10.1128/mcb.18.1.260 PMC121488

[9] Moreau S Ferguson JR Symington LS 1999 The nuclease activity of Mre11 is required for meiosis but not for mating type switching, end joining, or telomere maintenance. Mol. Cell Biol. 19, 556–566 9858579 10.1128/mcb.19.1.556 PMC83913

[10] Tsubouchi H Ogawa H 2000 Exo1 roles for repair of DNA double-strand breaks and meiotic crossing over in *Saccharomyces cerevisiae*. Mol. Biol. Cell 11, 2221–2233 (doi:10.1091/mbc.11.7.2221) 10888664 10.1091/mbc.11.7.2221 PMC14915

[11] Neale MJ Pan J Keeney S 2005 Endonucleolytic processing of covalent protein-linked DNA double-strand breaks. Nature 436, 1053–1057 (doi:10.1038/nature03872) 16107854 10.1038/nature03872 PMC1262668

[12] Hartsuiker E Mizuno K Molnar M Kohli J Ohta K Carr AM 2009 Ctp1CtIP and Rad32Mre11 nuclease activity are required for Rec12Spo11 removal, but Rec12Spo11 removal is dispensable for other MRN-dependent meiotic functions. Mol. Cell Biol. 29, 1671–1681 (doi:10.1128/MCB.01182-08) 19139281 10.1128/MCB.01182-08 PMC2655602

[13] Milman N Higuchi E Smith GR 2009 Meiotic DNA double-strand break repair requires two nucleases, MRN and Ctp1, to produce a single size class of Rec12 (Spo11)-oligonucleotide complexes. Mol. Cell Biol. 29, 5998–6005 (doi:10.1128/MCB.01127-09) 19752195 10.1128/MCB.01127-09 PMC2772569

[14] Rothenberg M Kohli J Ludin K 2009 Ctp1 and the MRN-complex are required for endonucleolytic Rec12 removal with release of a single class of oligonucleotides in fission yeast. PLoS Genet. 5, e1000722 (doi:10.1371/journal.pgen.1000722) 19911044 10.1371/journal.pgen.1000722 PMC2768786

[15] Zakharyevich K Ma Y Tang S Hwang PY Boiteux S Hunter N 2010 Temporally and biochemically distinct activities of Exo1 during meiosis: double-strand break resection and resolution of double holliday junctions. Mol. Cell 40, 1001–1015 (doi:10.1016/j.molcel.2010.11.032) 21172664 10.1016/j.molcel.2010.11.032 PMC3061447

[16] Keelagher RE Cotton VE Goldman AS Borts RH 2010 Separable roles for Exonuclease I in meiotic DNA double-strand break repair. DNA Repair (Amst) 10, 126–173 (doi:10.1016/j.dnarep.2010.09.024) 21044871 10.1016/j.dnarep.2010.09.024

[17] Hodgson A Terentyev Y Johnson RA Bishop-Bailey A Angevin T Croucher A Goldman AS 2010 Mre11 and Exo1 contribute to the initiation and processivity of resection at meiotic double-strand breaks made independently of Spo11. DNA Repair (Amst) 10, 138–148 (doi:10.1016/j.dnarep.2010.11.008) 21146476 10.1016/j.dnarep.2010.11.008

[18] Garcia V Phelps SEL Gray S Neale MJ 2011 Bidirectional resection of DNA double-strand breaks by Mre11 and Exo1. Nature 479, 241–244 (doi:10.1038/nature10515) 22002605 10.1038/nature10515 PMC3214165

[19] Wold MS 1997 Replication protein A: a heterotrimeric, single-stranded DNA-binding protein required for eukaryotic DNA metabolism. Annu. Rev. Biochem. 66, 61–92 (doi:10.1146/annurev.biochem.66.1.61) 9242902 10.1146/annurev.biochem.66.1.61

[20] Keeney S Kleckner N 1995 Covalent protein-DNA complexes at the 5′ strand termini of meiosis-specific double-strand breaks in yeast. Proc. Natl Acad. Sci. USA 92, 11 274–11 278 (doi:10.1073/pnas.92.24.11274) 10.1073/pnas.92.24.11274 PMC40614 7479978

[21] Liu J Wu TC Lichten M 1995 The location and structure of double-strand DNA breaks induced during yeast meiosis: evidence for a covalently linked DNA-protein intermediate. EMBO J. 14, 4599–4608 7556103 10.1002/j.1460-2075.1995.tb00139.x PMC394552

[22] Bishop DK Park D Xu L Kleckner N 1992 DMC1: a meiosis-specific yeast homolog of *E. coli recA* required for recombination, synaptonemal complex formation, and cell cycle progression. Cell 69, 439–456 (doi:10.1016/0092-8674(92)90446-J) 1581960 10.1016/0092-8674(92)90446-j

[23] Shinohara A Ogawa H Ogawa T 1992 Rad51 protein involved in repair and recombination in *S. cerevisiae* is a RecA-like protein. Cell 69, 457–470 (doi:10.1016/0092-8674(92)90447-K) 1581961 10.1016/0092-8674(92)90447-k

[24] Lydall D Nikolsky Y Bishop DK Weinert T 1996 A meiotic recombination checkpoint controlled by mitotic checkpoint genes. Nature 383, 840–843 (doi:10.1038/383840a0) 8893012 10.1038/383840a0

[25] Tung KS Hong EJ Roeder GS 2000 The pachytene checkpoint prevents accumulation and phosphorylation of the meiosis-specific transcription factor Ndt80. Proc. Natl Acad. Sci. USA 97, 12 187–12 192 (doi:10.1073/pnas.220464597) 11035815 10.1073/pnas.220464597 PMC17316

[26] Yoshida K Kondoh G Matsuda Y Habu T Nishimune Y Morita T 1998 The mouse RecA-like gene Dmc1 is required for homologous chromosome synapsis during meiosis. Mol. Cell 1, 707–718 (doi:10.1016/S1097-2765(00)80070-2) 9660954 10.1016/s1097-2765(00)80070-2

[27] Pittman DL Cobb J Schimenti KJ Wilson LA Cooper DM Brignull E Handel MA Schimenti JC 1998 Meiotic prophase arrest with failure of chromosome synapsis in mice deficient for Dmc1, a germline-specific RecA homolog. Mol. Cell 1, 697–705 (doi:10.1016/S1097-2765(00)80069-6) 9660953 10.1016/s1097-2765(00)80069-6

[28] Bermudez VP Lindsey-Boltz LA Cesare AJ Maniwa Y Griffith JD Hurwitz J Sancar A 2003 Loading of the human 9-1-1 checkpoint complex onto DNA by the checkpoint clamp loader hRad17-replication factor C complex in vitro. Proc. Natl Acad. Sci. USA 100, 1633–1638 (doi:10.1073/pnas.0437927100) 12578958 10.1073/pnas.0437927100 PMC149884

[29] Zou L Liu D Elledge SJ 2003 Replication protein A-mediated recruitment and activation of Rad17 complexes. Proc. Natl Acad. Sci. USA 100, 13 827–13 832 (doi:10.1073/pnas.2336100100) 10.1073/pnas.2336100100 PMC283506 14605214

[30] Zou L Elledge SJ 2003 Sensing DNA damage through ATRIP recognition of RPA-ssDNA complexes. Science 300, 1542–1548 (doi:10.1126/science.1083430) 12791985 10.1126/science.1083430

[31] Zou L 2007 Single- and double-stranded DNA: building a trigger of ATR-mediated DNA damage response. Genes Dev. 21, 879–985 (doi:10.1101/gad.1550307) 17437994 10.1101/gad.1550307

[32] Majka J Burgers PM 2003 Yeast Rad17/Mec3/Ddc1: a sliding clamp for the DNA damage checkpoint. Proc. Natl Acad. Sci. USA 100, 2249–2254 (doi:10.1073/pnas.0437148100) 12604797 10.1073/pnas.0437148100 PMC151326

[33] Majka J Niedziela-Majka A Burgers PM 2006 The checkpoint clamp activates Mec1 kinase during initiation of the DNA damage checkpoint. Mol. Cell 24, 891–901 (doi:10.1016/j.molcel.2006.11.027) 17189191 10.1016/j.molcel.2006.11.027 PMC1850967

[34] Carballo JA Johnson AL Sedgwick SG Cha RS 2008 Phosphorylation of the axial element protein Hop1 by Mec1/Tel1 ensures meiotic interhomolog recombination. Cell 132, 758–770 (doi:10.1016/j.cell.2008.01.035) 18329363 10.1016/j.cell.2008.01.035

[35] Refolio E Cavero S Marcon E Freire R San-Segundo PA 2011 The Ddc2/ATRIP checkpoint protein monitors meiotic recombination intermediates. J. Cell Sci. 124, 2488–2500 (doi:10.1242/jcs.081711) 21693576 10.1242/jcs.081711

[36] Sanchez Y Bachant J Wang H Hu F Liu D Tetzlaff M Elledge SJ 1999 Control of the DNA damage checkpoint by Chk1 and Rad53 protein kinases through distinct mechanisms. Science 286, 1166–1171 (doi:10.1126/science.286.5442.1166) 10550056 10.1126/science.286.5442.1166

[37] Roeder GS Bailis JM 2000 The pachytene checkpoint. Trends Genet. 16, 395–403 (doi:10.1016/S0168-9525(00)02080-1) 10973068 10.1016/s0168-9525(00)02080-1

[38] Usui T Ogawa H Petrini JH 2001 A DNA damage response pathway controlled by Tel1 and the Mre11 complex. Mol. Cell 7, 1255–1266 (doi:10.1016/S1097-2765(01)00270-2) 11430828 10.1016/s1097-2765(01)00270-2

[39] Wan L de los Santos T Zhang C Shokat K Hollingsworth NM 2004 Mek1 kinase activity functions downstream of *RED1* in the regulation of meiotic double strand break repair in budding yeast. Mol. Biol. Cell 15, 11–23 (doi:10.1091/mbc.E03-07-0499) 14595109 10.1091/mbc.E03-07-0499 PMC307523

[40] Shinohara M Sakai K Ogawa T Shinohara A 2003 The mitotic DNA damage checkpoint proteins Rad17 and Rad24 are required for repair of double-strand breaks during meiosis in yeast. Genetics 164, 855–865 12871899 10.1093/genetics/164.3.855 PMC1462628

[41] Grushcow JM Holzen TM Park KJ Weinert T Lichten M Bishop DK 1999 *Saccharomyces cerevisiae* checkpoint genes *MEC1*, *RAD17* and *RAD24* are required for normal meiotic recombination partner choice. Genetics 153, 607–620 10511543 10.1093/genetics/153.2.607 PMC1460798

[42] Xu L Weiner BM Kleckner N 1997 Meiotic cells monitor the status of the interhomolog recombination complex. Genes Dev. 11, 106–118 (doi:10.1101/gad.11.1.106) 9000054 10.1101/gad.11.1.106

[43] Niu H Li X Job E Park C Moazed D Gygi SP Hollingsworth NM 2007 Mek1 kinase is regulated to suppress double-strand break repair between sister chromatids during budding yeast meiosis. Mol. Cell Biol. 27, 5456–5467 (doi:10.1128/MCB.00416-07) 17526735 10.1128/MCB.00416-07 PMC1952091

[44] Terentyev Y Johnson R Neale MJ Khisroon M Bishop-Bailey A Goldman AS 2010 Evidence that *MEK1* positively promotes interhomologue double-strand break repair. Nucleic Acids Res. 38, 4349–4360 (doi:10.1093/nar/gkq137) 20223769 10.1093/nar/gkq137 PMC2910038

[45] Lange J Pan J Cole F Thelen MP Jasin M Keeney S 2011 ATM controls meiotic double-strand-break formation. Nature 479, 237–240 (doi:10.1038/nature10508) 22002603 10.1038/nature10508 PMC3213282

[46] Joyce EF Pedersen M Tiong S White-Brown SK Paul A Campbell SD McKim KS 2011 *Drosophila* ATM and ATR have distinct activities in the regulation of meiotic DNA damage and repair. J. Cell Biol. 195, 359–367 (doi:10.1083/jcb.201104121) 22024169 10.1083/jcb.201104121 PMC3206348

[47] Zhang L Kleckner NE Storlazzi A Kim KP 2011 Meiotic double-strand breaks occur once per pair of (sister) chromatids and, via Mec1/ATR and Tel1/ATM, once per quartet of chromatids. Proc. Natl Acad. Sci. USA 108, 20 036–20 041 (doi:10.1073/pnas.1117937108) 10.1073/pnas.1117937108 PMC3250133 22123968

[48] Pan J 2011 A hierarchical combination of factors shapes the genome-wide topography of yeast meiotic recombination initiation. Cell 144, 719–731 (doi:10.1016/j.cell.2011.02.009) 21376234 10.1016/j.cell.2011.02.009 PMC3063416

[49] Martini E Borde V Legendre M Audic S Regnault B Soubigou G Dujon B Llorente B Lichten M 2011 Genome-wide analysis of heteroduplex DNA in mismatch repair-deficient yeast cells reveals novel properties of meiotic recombination pathways. PLoS Genet. 7, e1002305 (doi:10.1371/journal.pgen.1002305) 21980306 10.1371/journal.pgen.1002305 PMC3183076

[50] Neale MJ Keeney S 2009 End-labeling and analysis of Spo11-oligonucleotide complexes in *Saccharomyces cerevisiae*. Methods Mol. Biol. 557, 183–195 (doi:10.1007/978-1-59745-527-5_12) 19799183 10.1007/978-1-59745-527-5_12 PMC3162315

[51] Martini E Diaz RL Hunter N Keeney S 2006 Crossover homeostasis in yeast meiosis. Cell 126, 285–295 (doi:10.1016/j.cell.2006.05.044) 16873061 10.1016/j.cell.2006.05.044 PMC1949389

[52] Cao L Alani E Kleckner N 1990 A pathway for generation and processing of double-strand breaks during meiotic recombination in *S. cerevisiae*. Cell 61, 1089–1101 (doi:10.1016/0092-8674(90)90072-M) 2190690 10.1016/0092-8674(90)90072-m

[53] Diaz RL Alcid AD Berger JM Keeney S 2002 Identification of residues in yeast Spo11p critical for meiotic DNA double-strand break formation. Mol. Cell Biol. 22, 1106–1115 (doi:10.1128/MCB.22.4.1106-1115.2002) 11809802 10.1128/MCB.22.4.1106-1115.2002 PMC134631

[54] Schwacha A Kleckner N 1994 Identification of joint molecules that form frequently between homologs but rarely between sister chromatids during yeast meiosis. Cell 76, 51–63 (doi:10.1016/0092-8674(94)90172-4) 8287479 10.1016/0092-8674(94)90172-4

[55] Collins I Newlon CS 1994 Meiosis-specific formation of joint DNA molecules containing sequences from homologous chromosomes. Cell 76, 65–75 (doi:10.1016/0092-8674(94)90173-2) 8287480 10.1016/0092-8674(94)90173-2

[56] Xu L Ajimura M Padmore R Klein C Kleckner N 1995 *NDT80*, a meiosis-specific gene required for exit from pachytene in *Saccharomyces cerevisiae*. Mol. Cell Biol. 15, 6572–6581 8524222 10.1128/mcb.15.12.6572 PMC230910

[57] Chu S Herskowitz I 1998 Gametogenesis in yeast is regulated by a transcriptional cascade dependent on Ndt80. Mol. Cell 1, 685–696 (doi:10.1016/S1097-2765(00)80068-4) 9660952 10.1016/s1097-2765(00)80068-4

[58] Benjamin KR Zhang C Shokat KM Herskowitz I 2003 Control of landmark events in meiosis by the CDK Cdc28 and the meiosis-specific kinase Ime2. Genes Dev. 17, 1524–1539 (doi:10.1101/gad.1101503) 12783856 10.1101/gad.1101503 PMC196082

[59] Clyne RK Katis VL Jessop L Benjamin KR Herskowitz I Lichten M Nasmyth K 2003 Polo-like kinase Cdc5 promotes chiasmata formation and cosegregation of sister centromeres at meiosis I. Nat. Cell Biol. 5, 480–485 (doi:10.1038/ncb977) 12717442 10.1038/ncb977

[60] Lee BH Amon A 2003 Role of Polo-like kinase *CDC5* in programming meiosis I chromosome segregation. Science 300, 482–486 (doi:10.1126/science.1081846) 12663816 10.1126/science.1081846

[61] Borde V Goldman AS Lichten M 2000 Direct coupling between meiotic DNA replication and recombination initiation. Science 290, 806–869 (doi:10.1126/science.290.5492.806) 11052944 10.1126/science.290.5492.806

[62] Henderson KA Kee K Maleki S Santini PA Keeney S 2006 Cyclin-dependent kinase directly regulates initiation of meiotic recombination. Cell 125, 1321–1332 (doi:10.1016/j.cell.2006.04.039) 16814718 10.1016/j.cell.2006.04.039 PMC1950680

[63] Wan L Niu H Futcher B Zhang C Shokat KM Boulton SJ Hollingsworth NM 2008 Cdc28-Clb5 (CDK-S) and Cdc7-Dbf4 (DDK) collaborate to initiate meiotic recombination in yeast. Genes Dev. 22, 386–397 (doi:10.1101/gad.1626408) 18245450 10.1101/gad.1626408 PMC2216697

[64] Sasanuma H Hirota K Fukuda T Kakusho N Kugou K Kawasaki Y Shibata T Masai H Ohta K 2008 Cdc7-dependent phosphorylation of Mer2 facilitates initiation of yeast meiotic recombination. Genes Dev. 22, 398–410 (doi:10.1101/gad.1626608) 18245451 10.1101/gad.1626608 PMC2216698

[65] Borde V Robine N Lin W Bonfils S Geli V Nicolas A 2009 Histone H3 lysine 4 trimethylation marks meiotic recombination initiation sites. EMBO J. 28, 99–111 (doi:10.1038/emboj.2008.257) 19078966 10.1038/emboj.2008.257 PMC2634730

[66] Neale MJ 2010 PRDM9 points the zinc finger at meiotic recombination hotspots. Genome Biol. 11, 104 (doi:10.1186/gb-2010-11-2-104) 20210982 10.1186/gb-2010-11-2-104 PMC2872867

[67] Panizza S Mendoza MA Berlinger M Huang L Nicolas A Shirahige K Klein F 2011 Spo11-accessory proteins link double-strand break sites to the chromosome axis in early meiotic recombination. Cell 146, 372–383 (doi:10.1016/j.cell.2011.07.003) 21816273 10.1016/j.cell.2011.07.003

[68] Hepworth SR Friesen H Segall J 1998 *NDT80* and the meiotic recombination checkpoint regulate expression of middle sporulation-specific genes in *Saccharomyces cerevisiae*. Mol. Cell Biol. 18, 5750–5761 9742092 10.1128/mcb.18.10.5750 PMC109161

[69] Kim ST Lim DS Canman CE Kastan MB 1999 Substrate specificities and identification of putative substrates of ATM kinase family members. J. Biol. Chem. 274, 37 538–37 543 (doi:10.1074/jbc.274.53.37538) 10.1074/jbc.274.53.37538 10608806

[70] Carballo JA Lichten M Panizza S Serrentino ME Johnson AL Geymonat M Borde V Klein F Cha RS 2013 Budding yeast ATM/ATR control meiotic double-strand break (DSB) levels by down-regulating Rec114, an essential component of the DSB-machinery. PLoS Genet. 9, e1003545 (doi:10.1371/journal.pgen.1003545) 23825959 10.1371/journal.pgen.1003545 PMC3694840

[71] Li J Hooker GW Roeder GS 2006 *Saccharomyces cerevisiae* Mer2, Mei4 and Rec114 form a complex required for meiotic double-strand break formation. Genetics 173, 1969–1981 (doi:10.1534/genetics.106.058768) 16783010 10.1534/genetics.106.058768 PMC1569690

[72] Maleki S Neale MJ Arora C Henderson KA Keeney S 2007 Interactions between Mei4, Rec114, and other proteins required for meiotic DNA double-strand break formation in *Saccharomyces cerevisiae*. Chromosoma 116, 471–486 (doi:10.1007/s00412-007-0111-y) 17558514 10.1007/s00412-007-0111-y PMC2084462

[73] Kauppi L Barchi M Lange J Baudat F Jasin M Keeney S 2013 Numerical constraints and feedback control of double-strand breaks in mouse meiosis. Genes Dev. 27, 873–886 (doi:10.1101/gad.213652.113) 23599345 10.1101/gad.213652.113 PMC3650225

[74] Xu L Kleckner N 1995 Sequence non-specific double-strand breaks and interhomolog interactions prior to double-strand break formation at a meiotic recombination hot spot in yeast. EMBO J. 14, 5115–5128 7588640 10.1002/j.1460-2075.1995.tb00194.x PMC394615

[75] Mancera E Bourgon R Brozzi A Huber W Steinmetz LM 2008 High-resolution mapping of meiotic crossovers and non-crossovers in yeast. Nature 454, 479–485 (doi:10.1038/nature07135) 18615017 10.1038/nature07135 PMC2780006

[76] Cole F Kauppi L Lange J Roig I Wang R Keeney S Jasin M 2012 Homeostatic control of recombination is implemented progressively in mouse meiosis. Nat. Cell Biol. 14, 424–430 (doi:10.1038/ncb2451) 22388890 10.1038/ncb2451 PMC3319518

[77] Abdullah MF Borts RH 2001 Meiotic recombination frequencies are affected by nutritional states in *Saccharomyces cerevisiae*. Proc. Natl Acad. Sci. USA 98, 14 524–14 529 (doi:10.1073/pnas.201529598) 11724920 10.1073/pnas.201529598 PMC64715

[78] Borner GV Kleckner N Hunter N 2004 Crossover/noncrossover differentiation, synaptonemal complex formation, and regulatory surveillance at the leptotene/zygotene transition of meiosis. Cell 117, 29–45 (doi:10.1016/S0092-8674(04)00292-2) 15066280 10.1016/s0092-8674(04)00292-2

[79] Chan AC Borts RH Hoffmann E 2009 Temperature-dependent modulation of chromosome segregation in *msh4* mutants of budding yeast. PLoS ONE 4, e7284 (doi:10.1371/journal.pone.0007284) 19816584 10.1371/journal.pone.0007284 PMC2757900

[80] Grandin N Reed SI 1993 Differential function and expression of *Saccharomyces cerevisiae* B-type cyclins in mitosis and meiosis. Mol. Cell Biol. 13, 2113–2125 8455600 10.1128/mcb.13.4.2113-2125.1993 PMC359532

[81] Jessop L Rockmill B Roeder GS Lichten M 2006 Meiotic chromosome synapsis-promoting proteins antagonize the anti-crossover activity of Sgs1. PLoS Genet. 2, e155 (doi:10.1371/journal.pgen.0020155) 17002499 10.1371/journal.pgen.0020155 PMC1570379

[82] Murakami H Borde V Nicolas A Keeney S 2009 Gel electrophoresis assays for analyzing DNA double-strand breaks in *Saccharomyces cerevisiae* at various spatial resolutions. Methods Mol. Biol. 557, 117–142 (doi:10.1007/978-1-59745-527-5_9) 19799180 10.1007/978-1-59745-527-5_9 PMC3157973

