# Supplementary material for: Positive regulation of meiotic DNA double-strand break formation by activation of the DNA damage checkpoint kinase Mec1(ATR)
Source: Open Biol. 2013 Jul;3(7):130019. doi: 10.1098/rsob.130019 (PMC3728922; doi:10.1098/rsob.130019)
Supplement: Supplementary table and supplementary figures 1-6 [file rsob130019-s1.pdf]

Supplementary Table 1 – *S. cerevisiae* strains used in this study (in order of appearance)

Strain	Number	Genotype
Wildtype	MJ6	*ho::LYS2/', lys2/', ura3/', arg4-nsp/', leu2::hisG/', his4X::LEU2/', nuc1::LEU2/'*
spo11-HA	MJ13	*ho::LYS2/', lys2/', ura3/', arg4-nsp/', leu2::hisG/', his4X::LEU2/', nuc1::LEU2/', SPO11-HA3His6::KanMX4/'*
dmc1Δ	SG147	*ho::LYS2/', lys2/', ura3/', arg4-nsp/', leu2::hisG/', his4X::LEU2/', nuc1::LEU2/', dmc1Δ::LEU2/'*
dmc1Δ spo11-HA	SG32	*ho::LYS2/', lys2/', ura3/', arg4-Nsp/', leu2::hisG/', his4X::LEU2/', nuc1::LEU2/', dmc1Δ::LEU2/', SPO11-HA3His6::KanMX4/'*
rad24Δ	SG150	*ho::LYS2/', lys2/', ura3/', arg4-nsp/', leu2::hisG/', his4X::LEU2/', nuc1::LEU2/', rad24Δ::hpHMX4/'*
rad24Δ spo11-HA	SG69	*ho::LYS2/', lys2/', ura3/', arg4-nsp/', leu2::hisG/', his4X::LEU2/', nuc1::LEU2/', rad24Δ::hpHMX4/', SPO11-HA3His6::KanMX4/'*
rad24Δ dmc1Δ	SG146	*ho::LYS2/', lys2/', ura3/', arg4-nsp/', leu2::hisG/', his4X::LEU2/', nuc1::LEU2/', dmc1Δ::LEU2/', rad24Δ::hpHMX4/'*
rad24Δ dmc1Δ spo11-HA	SG29	*ho::LYS2/', lys2/', ura3/', arg4-nsp/', leu2::hisG/', his4X::LEU2/', nuc1::LEU2/', dmc1Δ::LEU2/',rad24Δ::hpHMX4/', SPO11-HA3His6::KanMX4/'*
rad24Δ rad51Δ dmc1Δ	SG243	*ho::hisG/', lys2/', ura3/', arg4-nsp/', leu2::hisG/', his4X::LEU2/', nuc1::LEU2/', dmc1Δ::LEU2/', rad24Δ::hpHMX4/', rad51Δ::hisG-URA3-hisG/'*
rad24Δ rad51Δ dmc1Δ spo11-HA	SG242	*ho::LYS2/', lys2/', ura3/', arg4-nsp/', leu2::hisG/', his4X::LEU2/', nuc1::LEU2/', dmc1Δ::LEU2/', rad24Δ::hpHMX4/', rad51Δ::hisG-URA3-hisG/', SPO11-HA3His6::KanMX4/'*
ndt80Δ rad24Δ rad51Δ dmc1Δ spo11-HA	SG406	*ho::LYS2/', lys2/', ura3/', leu2::hisG/', his3-?/', his4X::LEU2/', nuc1::LEU2/', dmcΔ::LEU2/', rad24Δ::hpHMX4/', rad51Δ::hisG-URA3-hisG/', SPO11-HA3His6::KanMX4/', ndt80Δ::hphMX4/'*
sae2Δ	MJ315	*ho::LYS2/', lys2/', ura3/', arg4-nsp/', leu2::hisG/', his4X::LEU2/', nuc1::LEU2/', sae2Δ::KanMX/'*
sae2Δ spo11-HA	MJ10	*ho::LYS2/', lys2/', ura3/', arg4-nsp/', leu2::hisG/', his4X::LEU2/', nuc1::LEU2/', sae2Δ::KanMX/', SPO11-HA3His6::KanMX4/'*
sae2Δ rad24Δ	SG103	*ho::LYS2/', lys2/', ura3/', arg4-nsp/', leu2::hisG/', his4X::LEU2/', nuc1::LEU2/', rad24Δ::hpHMX4/', sae2Δ::KanMX/'*
sae2Δ rad24Δ spo11-HA	SG106	*ho::LYS2/', lys2/', ura3/', arg4-nsp/', leu2::hisG/', his4X::LEU2/', nuc1::LEU2/', rad24Δ::hpHMX4/', sae2Δ::KanMX/', SPO11-HA3His6::KanMX4/'*
pCLB2-MEC1	SG286	*ho::LYS2/', lys2/', ura3/', arg4-nsp/', leu2::hisG/', his4X::LEU2/', nuc1::LEU2/', ade2-Bgl/ADE2, pCLB2-MEC1::KanMX/'*
pCLB2-MEC1 spo11-HA	SG261	*ho::LYS2/', lys2/', ura3/', arg4-nsp/', leu2::hisG/', his4X::LEU2/', nuc1::LEU2/', ade2-Bgl/ADE2, pCLB2-MEC1::KanMX4/', SPO11-HA3His6::KanMX4/'*
pCLB2-MEC1 dmc1Δ	SG283	*ho::LYS2/', lys2/', ura3/', arg4-nsp/', leu2::hisG/', his4X::LEU2/', nuc1::LEU2/', pCLB2-MEC1::KanMX/', dmc1Δ::HphMX/'*
pCLB2-MEC1 dmc1Δ spo11-HA	SG258	*ho::LYS2/', lys2/', ura3/', arg4-nsp/', leu2::hisG/', his4X::LEU2/', nuc1::LEU2/', pCLB2-MEC1::KanMX4/', dmc1Δ::HphMX/', SPO11-HA3His6::KanMX4/'*
rad17Δ	SG181	*ho::LYS2/', lys2/', ura3/', leu2::hisG/', his3-?/', his4X::LEU2/', nuc1Δ::LEU2/', rad17Δ::natMX/'*
rad17Δ spo11-HA	SG187	*ho::LYS2/', lys2/', ura3/', arg4-nsp/ARG4, leu2::hisG/', his3-?/', his4X::LEU2/', nuc1::LEU2/', rad17Δ::natMX/', SPO11-HA3His6::KanMX4/'*
rad17Δ dmc1Δ	SG177	*ho::LYS2/', lys2/', ura3/', arg4-nsp/ARG4, leu2::hisG, his3-?/', his4X::LEU2/', nuc1::LEU2/', rad17Δ::natMX/', dmc1Δ::LEU2/'*
rad17Δ dmc1Δ spo11-HA	SG180	*ho::LYS2/', lys2/', ura3/', arg4-nsp/', leu2::hisG/', his3-?/', his4X::LEU2/', nuc1::LEU2/', rad17Δ::natMX/', dmc1Δ::LEU2/', SPO11-HA3His6::KanMX4/'*
dmc1Δ spo11-D290A	MJ881	*ho::LYS2/', lys2/', ura3/', arg4-nsp/', leu2-?/', nuc1::LEU2/', his4B/X::LEU2/', dmc1Δ::hphMX/', spo11(D290A)::kanMX4/'*
dmc1Δ rad24Δ spo11-D290A	MJ882	*ho::LYS2/', lys2/', ura3/', arg4-nsp/', leu2-?/', nuc1::LEU2/', his4B/X::LEU2/', dmc1Δ::LEU2/', rad24Δ::hphMX/', spo11(D290A)::kanMX4/'*
sae2Δ spo11-D290A	MJ885	*ho::LYS2/', lys2/', ura3/', arg4-nsp/', leu2-?/', nuc1::LEU2/', his4-B/X?::LEU2/', sae2Δ::kanMX4/', spo11(D290A)::kanMX4/'*
dmc1Δ rad24Δ pCLB2-CDC5	SG460	*ho::LYS2/', lys2/', ura3/', leu2::hisG/', his4X::LEU2/', nuc1::LEU2/', dmc1Δ::LEU2/', rad24Δ::Hyg/', cdc5::pCLB2-CDC5::kanMX6/'*
dmc1Δ rad24Δ pCLB2-CDC5 spo11-HA	SG454	*ho::LYS2/', lys2/', ura3/', leu2::hisG/', his4X::LEU2/', nuc1::LEU2/', dmc1Δ::LEU2/', rad24Δ::Hyg/', cdc5::pCLB2-CDC5::kanMX6/', SPO11-HA3His6::KanMX4/'*
spo11-D290A	MJ878	*ho::LYS2/', lys2/', ura3/', arg4-nsp/', leu2-?/', nuc1::LEU2/', his4B/X::LEU2/', spo11(D290A)::kanMX4/'*
rad24Δ spo11-D290A	MJ879	*ho::LYS2/', lys2/', ura3/', arg4-nsp/', leu2-?/', nuc1::LEU2/', his4B/X::LEU2/', rad24Δ::hphMX/', spo11(D290A)::kanMX4/'*
pGAL-NDT80	MJ846	*ho::LYS2/", lys2/', arg4-nsp/', leu2::hisG/', trp1::hisG/', his4X::LEU2/', nuc1::LEU2/', ura3::PGPD1GAL4(848)-ER::URA3/', pGAL-NDT80::TRP1/'*
pGAL-NDT80 spo11-HA	MJ847	*ho::LYS2/", lys2/', arg4-nsp/', leu2::hisG/', trp1::hisG/', his4X::LEU2/', nuc1::LEU2/', ura3::PGPD1GAL4(848)-ER::URA3/', pGAL-NDT80::TRP1/', SPO11-HA3His6::KanMX4/'*
pGAL-NDT80 spo11-D290A	MJ892	*ho::LYS2/', lys2/', ura3/', arg4-nsp/', leu2-?/', trp1::hisG/', his4B/X::LEU2/', nuc1::LEU2/', ura3::PGPD1GAL4(848)-ER::URA3/', PGAL1NDT80::TRP1/', spo11(D290A)::kanMX/'*
pGAL-NDT80 rad24Δ	MJ848	*ho::LYS2/", lys2/', arg4-nsp/', leu2::hisG/', trp1::hisG/', his4X::LEU2/', nuc1::LEU2/', ura3::PGPD1GAL4(848)-ER::URA3/', pGAL-NDT80::TRP1/', rad24Δ::hphMX/'*
pGAL-NDT80 rad24Δ spo11-HA	MJ850	*ho::LYS2/", lys2/', arg4-nsp/', leu2::hisG/', trp1::hisG/', his4X::LEU2/', nuc1::LEU2/', ura3::PGPD1GAL4(848)-ER::URA3/', pGAL-NDT80::TRP1/', rad24Δ::hphMX/', SPO11-HA3His6::KanMX4/'*
pGAL-NDT80 rad24Δ spo11-D290A	MJ913	*ho::LYS2/', lys2/', ura3/', arg4-nsp/', leu2-?/', trp1::hisG/', his4B/X::LEU2/', nuc1::LEU2/', ura3::PGPD1GAL4(848)-ER::URA3/', pGAL-NDT80::TRP1/', rad24Δ::hphMX/', spo11(D290A)::kanMX4/'*

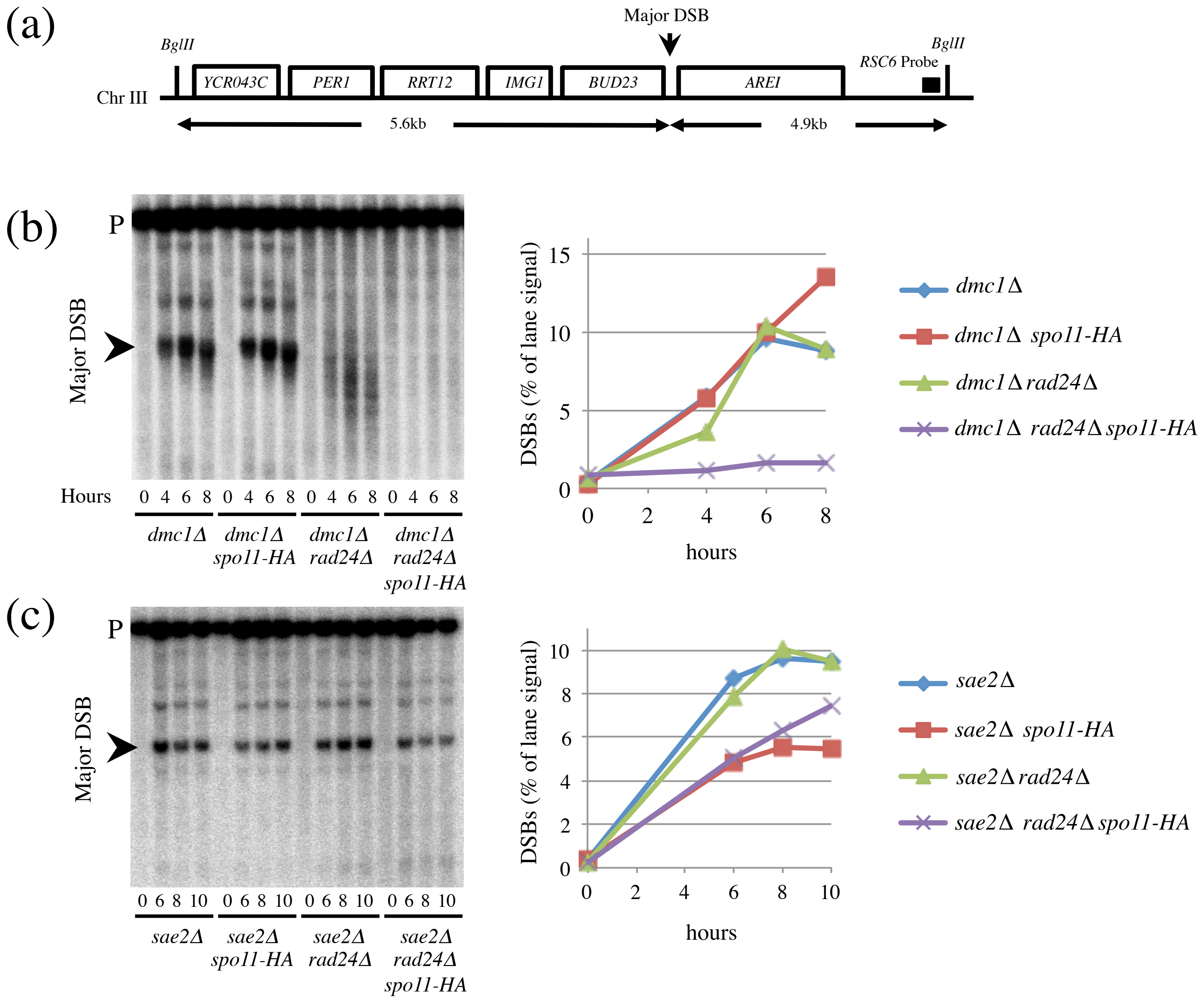

Figure S1. Analysis of DSB formation at the *ARE1* locus. a) Physical map of the *ARE1* region including *Bgl*II restriction sites, major DSB site and location of *RSC6* probe. b, c) Genomic DNA was isolated at the indicated timepoints from synchronous cultures of the indicated strains, digested with *Bgl*II, fractionated on a 0.7% agarose gel, transferred to nylon membrane, and hybridised with the *RSC6* probe. Arrowheads indicate DSB signals; P, parental band. Charts are quantification of the major DSB signal plotted as a percentage of total lane signal.

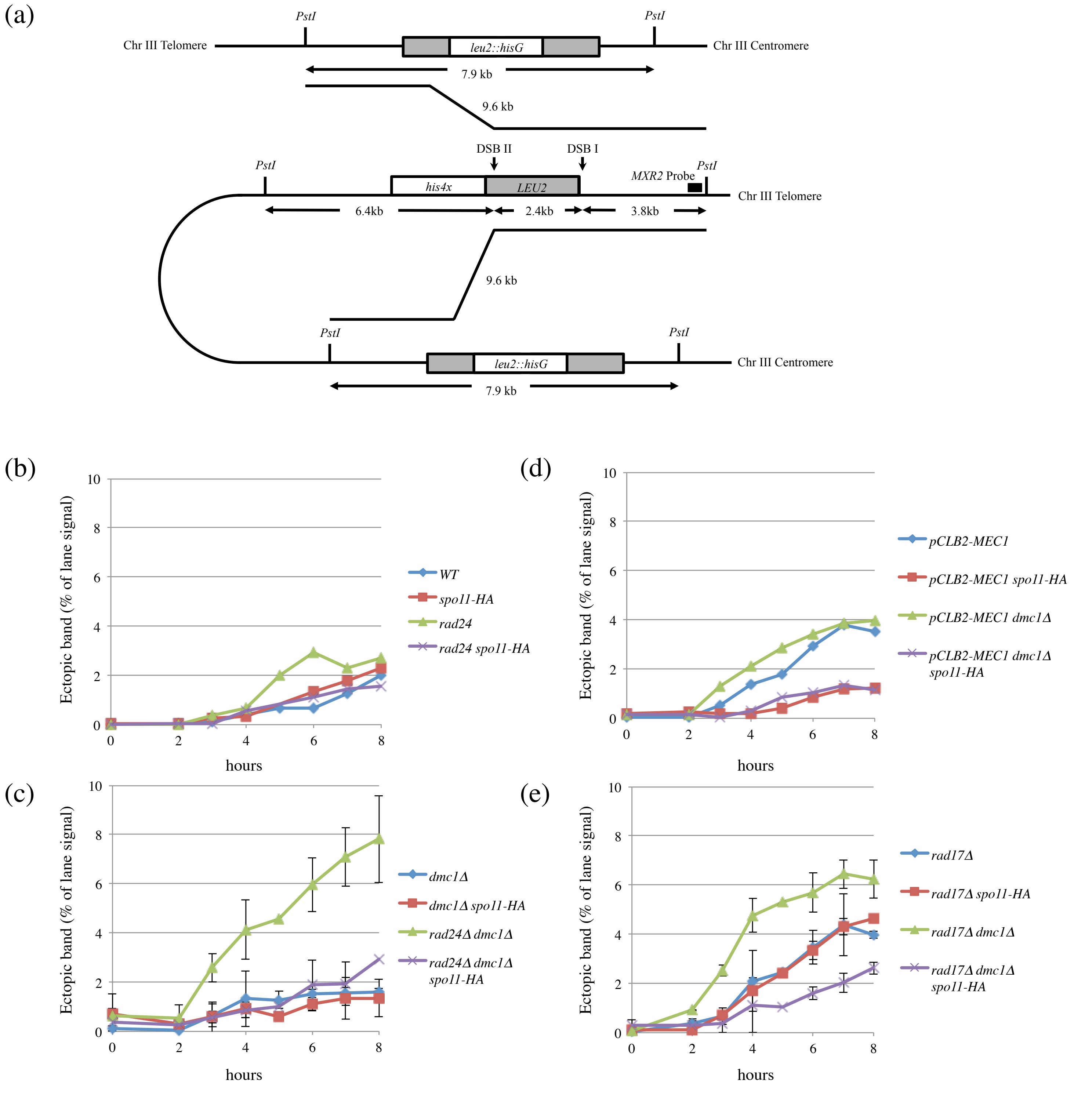

Figure S2. Quantification of ectopic recombination. a) Map of interchromatid (upper) and intrachromatid (lower) ectopic recombination events occurring between *HIS4*::*LEU2* and *leu2*::*hisG* [41]. b-e) Quantification of the ectopic band indicated in Fig 2b, 2e, 3a, 3c expressed as a percentage of total lane signal.

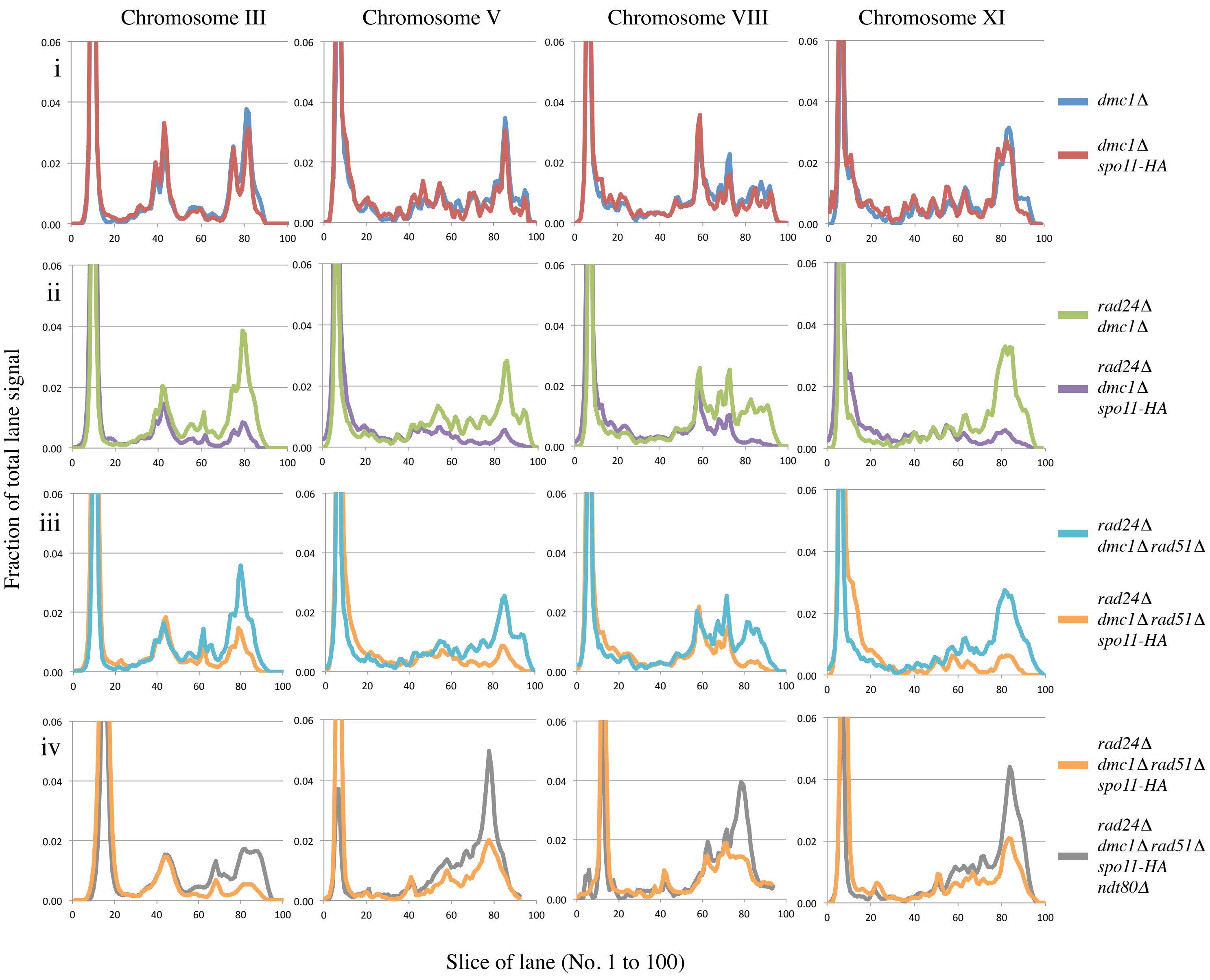

Figure S3. Comparison lane traces from select PFGE gels. Representative lane comparisons (panels i-iii and panel iv) for various strains/chromosomes for the gel images shown in Figure 2g and Figure 6c, respectively. Combined lane traces of 6-8 hours were exported from ImageGauge, resampled in Plot (OSX; Ver. 0.997) to create 100 equal lane slices, and displayed on the same scale as a fraction of total lane signal in each slice.

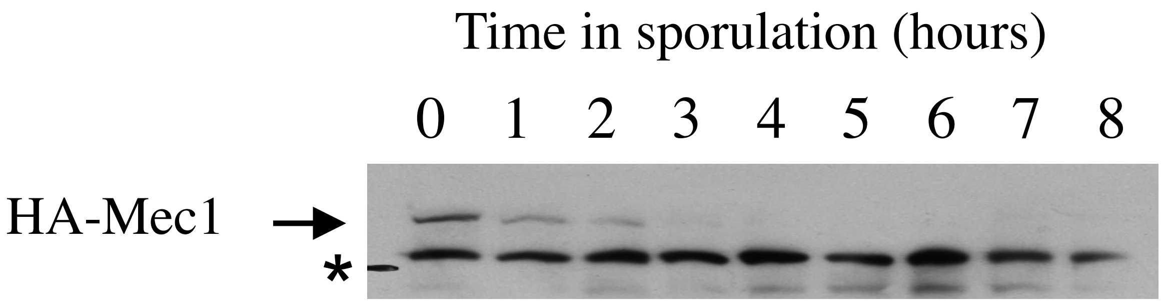

Figure S4. Replacement of the *MEC1* promoter with the *CLB2* promoter causes rapid loss of Mec1 protein during meiosis. The *CLB2* promoter was integrated in front of the *MEC1* gene which was N-terminal HA-epitope tagged at the same time. Western analysis using anti-HA antibody was used to detect HA-Mec1 protein throughout 8 hours of meiosis. While clearly present in pre-meiotic cells (0 h time point), the protein was difficult to detect from 3 h of meiosis, indicating that there was no meiotic expression of *MEC1* from the *CLB2* promoter. * Non-specific band.

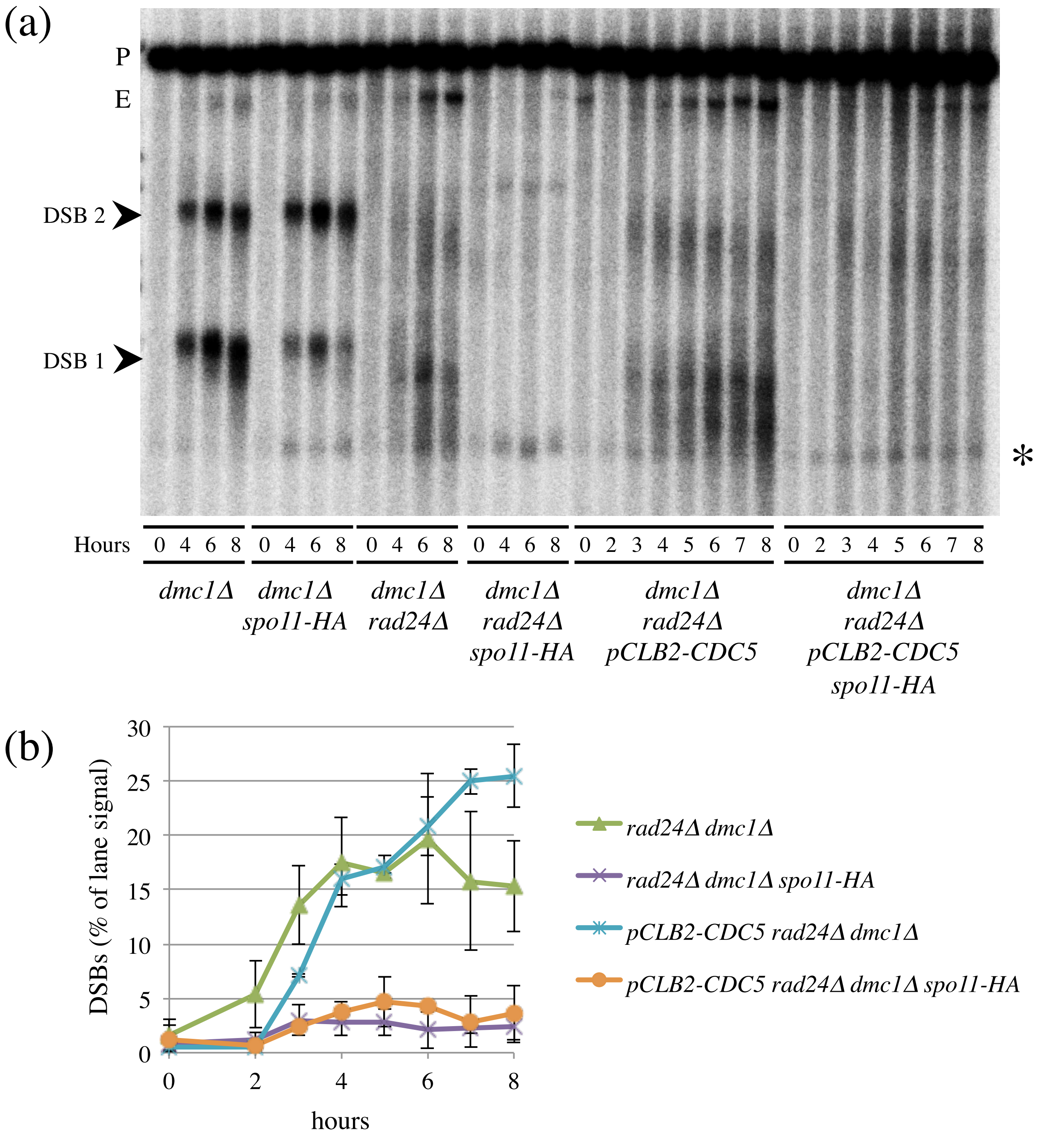

Figure S5. DSB formation in *rad24*Δ *dmc1*Δ *spo11-HA* is not rescued by Cdc5 depletion. a) Genomic DNA was isolated at the indicated timepoints from synchronous cultures of the indicated strains, digested with *Pst*I, fractionated on a 0.7% agarose gel, transferred to nylon membrane, and hybridised with the *MXR2* probe. Arrowheads indicate DSB signals, asterisk marks nonspecific band; P, parental band, E, ectopic band. b) Quantification of the total DSB signal (DSB 1 + DSB 2) shown in (a) plotted as a percentage of total lane signal. Plotted data are aggregated from multiple experiments.

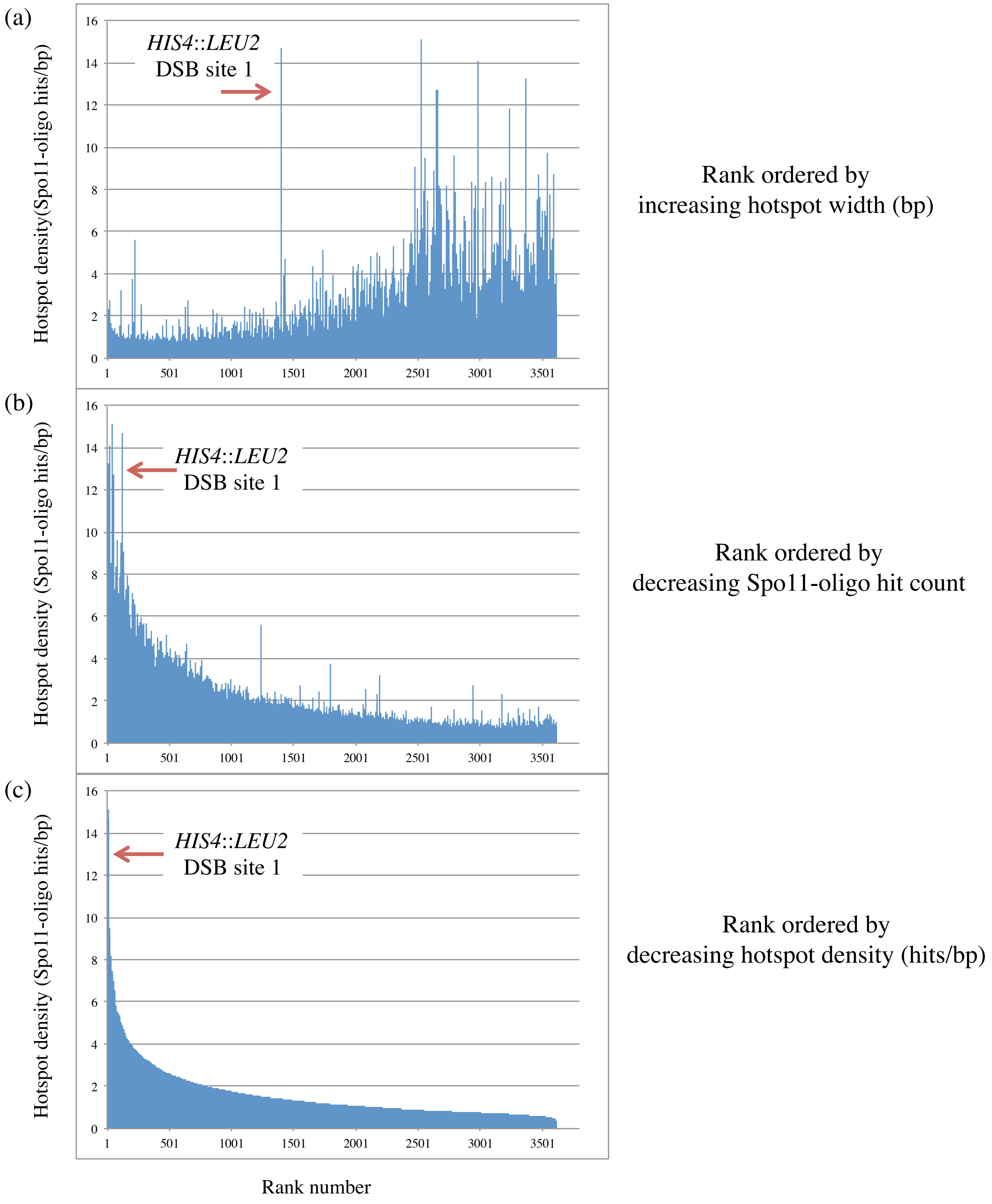

Figure S6. Analysis of Spo11-oligo hit count density at *HIS4*::*LEU2* DSB site 1. Spo11-oligo hit count data was used to calculate the relative density of Spo11-oligos per bp for the ~3600 annotated DSB hotspots [48]. We then rank ordered this data relative to: a) Increasing hotspot width in base pairs; b) Decreasing Spo11-oligo hit count; c) Decreasing hotspot density (hits/bp). The *HIS4*::*LEU2* DSB site 1 hotspot is indicated with a red arrow. It should be noted that these data are collated from experiments performed with the Spo11-HA3-his6 allele [48], which we show in this manuscript (figure 2b-f) to have a reduced frequency of DSBs at site 1 relative to in an untagged, *SPO11*$^{+}$, strain. It is probable, therefore, that the calculated density of DSBs at *HIS4*::*LEU2* site 1 is in fact an underestimate of the value that would be measured in wildtype (*SPO11*$^{+}$) cells. However it is also possible that the HA-tag also modulates hotspot width (leading to an associated increase or decrease in measured density). Future comparative analysis of different *SPO11* alleles will be informative to test these ideas further.
